# LinG3D: visualizing the spatio-temporal dynamics of clonal evolution

**DOI:** 10.1186/s12859-024-05813-7

**Published:** 2024-05-27

**Authors:** Anjun Hu, Awino Maureiq E. Ojwang’, Kayode D. Olumoyin, Katarzyna A. Rejniak

**Affiliations:** 1 Integrated Mathematical Oncology Department, H. Lee Moffitt Cancer Center and Research Institute, Tampa, FL 33612 USA; 2 Department of Chemistry, College of Arts and Sciences, University of South Florida, Tampa, FL 33612 USA; 3 Department of Oncologic Sciences, Morsani College of Medicine, University of South Florida, Tampa, FL 33612 USA

**Keywords:** Lineage trees, 3D visualization, Spatial clone distribution, Mathematical modeling of tumor evolution

## Abstract

**Background:**

Cancers are spatially heterogenous, thus their clonal evolution, especially following anti-cancer treatments, depends on where the mutated cells are located within the tumor tissue. For example, cells exposed to different concentrations of drugs, such as cells located near the vessels in contrast to those residing far from the vasculature, can undergo a different evolutionary path. However, classical representations of cell lineage trees do not account for this spatial component of emerging cancer clones. Here, we propose routines to trace spatial and temporal clonal evolution in computer simulations of the tumor evolution models.

**Results:**

The *LinG3D* (Lineage Graphs in 3D) is an open-source collection of routines (in MATLAB, Python, and R) that enables spatio-temporal visualization of clonal evolution in a two-dimensional tumor slice from computer simulations of the tumor evolution models. These routines draw traces of tumor clones in both time and space, and may include a projection of a selected microenvironmental factor, such as the drug or oxygen distribution within the tumor, if such a microenvironmental factor is used in the tumor evolution model. The utility of *LinG3D* has been demonstrated through examples of simulated tumors with different number of clones and, additionally, in experimental colony growth assay.

**Conclusions:**

This routine package extends the classical lineage trees, that show cellular clone relationships in time, by adding the space component to show the locations of cellular clones within the 2D tumor tissue patch from computer simulations of tumor evolution models.

**Supplementary Information:**

The online version contains supplementary material available at 10.1186/s12859-024-05813-7.

## Background

During tumor development, cells undergo somatic mutations that may offer them a survival advantage over other cells. This, in turn, can lead to the emergence of distinct cell populations, i.e., clones [1]. Tumor clonal diversity may be especially important when anti-cancer treatments are applied, since distinct clones can be affected differently, leaving those with a survival advantage, such as resistance to the drugs or radiotherapy, to persist. Thus, understanding how individual tumor clones emerge and spread may be vital in designing effective treatment therapies.

There are several ways to graphically present tumor clonal evolution. In one routinely used method, called “jellyfish” graphs or tumor evolution graphs, the horizontal axis represents the time along which plots with different colors indicate distinct genotypes. The height of each plot corresponds to that genotype’s relative abundance. If the clones generate descendant genotypes, they are shown as new areas emerging from inside their parents. These graphs showing changes in the sizes or frequencies of the clones over time can be drawn using the *ggmuller* [2], *fishplot* [3], or *evofreq* [4] routines. Another way to illustrate the development of tumor evolution is to show cell lineages in the form of a phylogenetic tree. In this graph, one starts with an initial cell (tree root) and connects each mother cell with its two daughter cells until all final descendants are reached (tree leaves). This produces a binary tree in which time or cell generations are represented by the axis joining the tree root with tree leaves, and the tree nodes are located at the times of the mother cells’ division. These graphs showing the dynamics of clone development can be drawn using the *phytree* [5], *ggtree* [6], or *TreeDyn* [7] routines.

However, the tumor clonal composition, such as the sizes and diversities of clones, depends strongly on the microenvironment surrounding the tumor. For example, cells located near the vessels are exposed to higher levels of bloodborne therapeutics and thus can be more prone to drug-induced death, thus generating smaller cellular clones, unless the cells develop resistance to that treatment. Therefore, they can undergo a different evolutionary path than cells residing far from vasculature, in areas with low drug levels where cells can proliferate more frequently and die less often, thus producing larger cellular clones or clones with more mutations. This spatial resolution is lacking in the classical clonal graphs, as those described above. We have designed the *LinG3D* routines by incorporating the space component to show the locations of cellular clones within the 2D patch of the tumor tissue, that is in relation to other cells, vasculature, or the distribution of drugs or nutrients. These routines are intended to visualize the 3D lineage trees for data generated by computational models of tumor evolution. However, they can be applied to experimental data, if appropriate information can be collected in the laboratory. In this paper, we describe four routines that form the *LinG3D* package allowing to visualize (i) an individual clone with all generated cells, (ii) an individual clone with no dead branches, i.e., the clone including only alive cells, (iii) all clones with all generated cells, and (iv) all clones with no dead branches. The utility of these routines is demonstrated using two examples of simulated tumors with different numbers of clones and an example of an experimental colony growth assay.

## Design and implementation

**Fig. 1:**
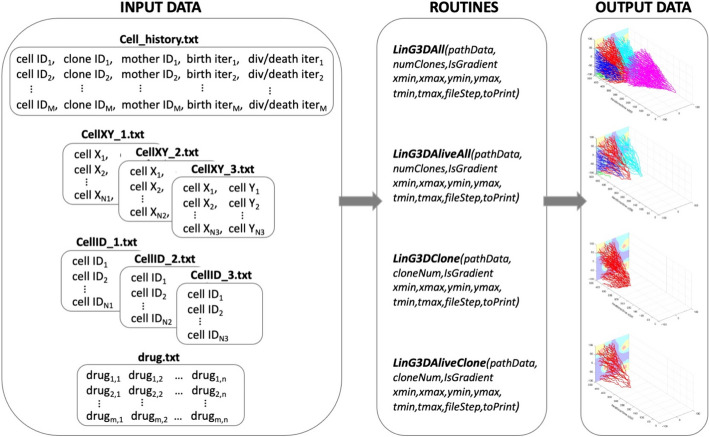
Architecture of the *LinG3D* routine package. The specified four types of text files are required as the input data for each of the four routines within the LinG3D package; each routine generates a graphical result as an output data

*LinG3D* has been created as a suite of routines that enable visualization of the spatio-temporal evolution of the cellular clones arising during computational simulations of mathematical models of tumor growth and/or its response to treatments. These routines display not only the mother cell-daughter cell linear relationship as in a classical lineage tree, but also show which subspace of the whole tumor tissue each clone occupies during its development and how these locations change over time. Four independent routines are provided to draw either an individual clone or all clones together, and with either all cells generated during the time of the computer simulation or the cells whose successors survived to the end of the simulation (i.e., by omitting branches with cells that died or left the simulation domain). The architecture of the four routines included in the *LinG3D* package is shown in Fig. 1.

The following routines are included in the *LinG3D* package.

### LinG3DAll

**Fig. 2:**
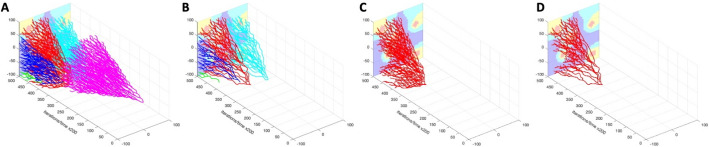
Examples of outcomes from four *LinG3D* routines. **A** a 3D full lineage tree with all cells and clones (*LinG3DAll* routine); **B** a 3D lineage tree with all surviving cells (*LinG3DAliveAll* routine); **C** a 3D lineage tree with all cells for a single clone (*LinG3DClone* routine); **D** a 3D lineage tree with all surviving cells in one clone (*LinG3DAliveClone* routine). In all examples, the drug distribution is shown in the background with concentrations from high to low represented by: red-yellow-cyan-blue

This routine draws a 3D spatio-temporal lineage tree taking into account all cells from all cellular clones. To do so, the routine starts with an initial cell (the tree root), and draws branches of the lineage tree for all mother-daughter cell pairs (straight line segments connecting these two cell positions), until the cells of each clone are included and the terminal cells with no descendants (clone leaves) are reached. An example of a full 3D lineage tree with five clones is shown in Fig. 2A. Routine syntax (all routine arguments are described below):

LinG3DAll(*pathData, numClones, IsGradient*,* xmin, xmax, ymin, ymax, tmin, tmax, fileStep, toPrint*).

### LinG3DAliveAll

This routine visualizes a 3D spatio-temporal lineage tree for all cellular clones, but includes only those cells for which at least one successor cell survived until the end of the simulation. Therefore, it omits branches with cells that died or that left the computational domain. The routine starts with cells in the last simulated iteration (tree leaves) and traces back all branches of the lineage tree for all daughter-mother cell pairs until the initial cell of the clone (the clone root) is reached. Figure 2B shows the 3D lineage tree of alive cells that corresponds to the full lineage tree from Fig. 2A. Routine syntax:

LinG3DAliveAll(*pathData, numClones, IsGradient*,* xmin, xmax, ymin, ymax, tmin, tmax, fileStep, toPrint*).

### LinG3DClone

This routine visualizes a 3D spatio-temporal lineage tree for one selected cellular clone showing all cells in that clone. This routine starts with the initial cell of the selected clone (the clone tree root) and draws all mother cell-daughter cell branches of the lineage tree for cells belonging to that clone until all cells are included and the terminal cells with no descendants (clone leaves) are reached. Figure 2C shows the full 3D lineage tree for one clone from Fig. 2A. Routine syntax:

*LinG3DClone*(*pathData, cloneNum, IsGradient*,* xmin, xmax, ymin, ymax, tmin, tmax, fileStep, toPrint*).

### LinG3DAliveClone

This routine draws a 3D spatio-temporal lineage tree for the surviving cells in the selected clone. The routine starts with the last iteration and selects cells that belong to the given clone (clone leaves). Next, it draws the branches for all daughter-mother cell pairs until the initial cell of that clone (the clone root) is reached. Figure 2D shows the 3D lineage tree for alive cells from one clone from Fig. 2B. Routine syntax:

*LinG3DAliveClone*(*pathData, cloneNum, IsGradient*,* xmin, xmax, ymin, ymax, tmin, tmax, fileStep, toPrint*).

In all routines described above, it is possible to rotate the graphs after they are generated using a computer mouse. This feature allows the user to find an angle upon which a particular cellular clone or clones under consideration will be visible in an optimal way.

#### Required format of the input data

All input data required by ***LinG3D*** routines consists of text files of four kinds. One text file contains information about the history of all cells to trace a given cell’s predecessors or successors, i.e., cell's index, the index of a clone it belongs to, the index of its mother cell, as well as the iteration numbers at which the cell was born, and at which it either divided or died. Moreover, for every iteration saved, two text files are required, one containing indices of all cells alive at this iteration, and the other with coordinates of cells present at this iteration. If the background gradient of the drug or metabolite should be drawn (*IsGradient* = 1), then a text file with a matrix of drug concentrations must be provided. Each of these text files is described in detail below.

The cell history data are saved in the following format, one cell per row (file in our code: *cell_history.txt*):

[*cell ID*, *clone ID*, *mother ID*, *birth iter*, *div/death iter*].

where columns contain *cell ID*—a unique ID number for the cell, *clone ID*—a number unique to a given clone to which the cell belongs, *mother ID*—a unique ID number of the cell’s mother cell, *birth iter*—the iteration number at which the cell was born, and *div/death iter*—the iteration number at which the cell either divided into two daughter cells or died. If the cell has not divided and is still alive, this element is equal to 0.

Moreover, for each iteration at which the lineage tree branches are drawn (denoted by *_#*), the following files are required to contain the x and y coordinates of all cells present at that iteration, one cell per row (file in our code: *cellXY_#.txt*):

[*cell X*, *cell Y*].

and the corresponding unique cell ID numbers, one cell per row (file in our code: *cellID_#.txt*):

[*cell ID*].

For every iteration saved (*_#*), the order of cells in these two files must be identical.

Additionally, if the microenvironmental factor distribution (such as oxygen or a drug) is to be drawn in the background, the file containing the factor concentrations per grid point should be saved as a 2D matrix (file in our code: *drug.txt*).

#### Expected output data

The output data generated by each of the ***LinG3D*** routines is an on-screen figure with a corresponding 3D lineage tree graph (Fig. 2A–D) showing clonal expansion in space (cell locations within the tumor tissue in the xz-plane) and in time (tumor clone’s temporal evolution along the y-axis). This figure can be automatically saved as a graphical file in the jpeg format, if *toPrint* = 1*.*

#### User-specified input function arguments

**Fig. 3:**
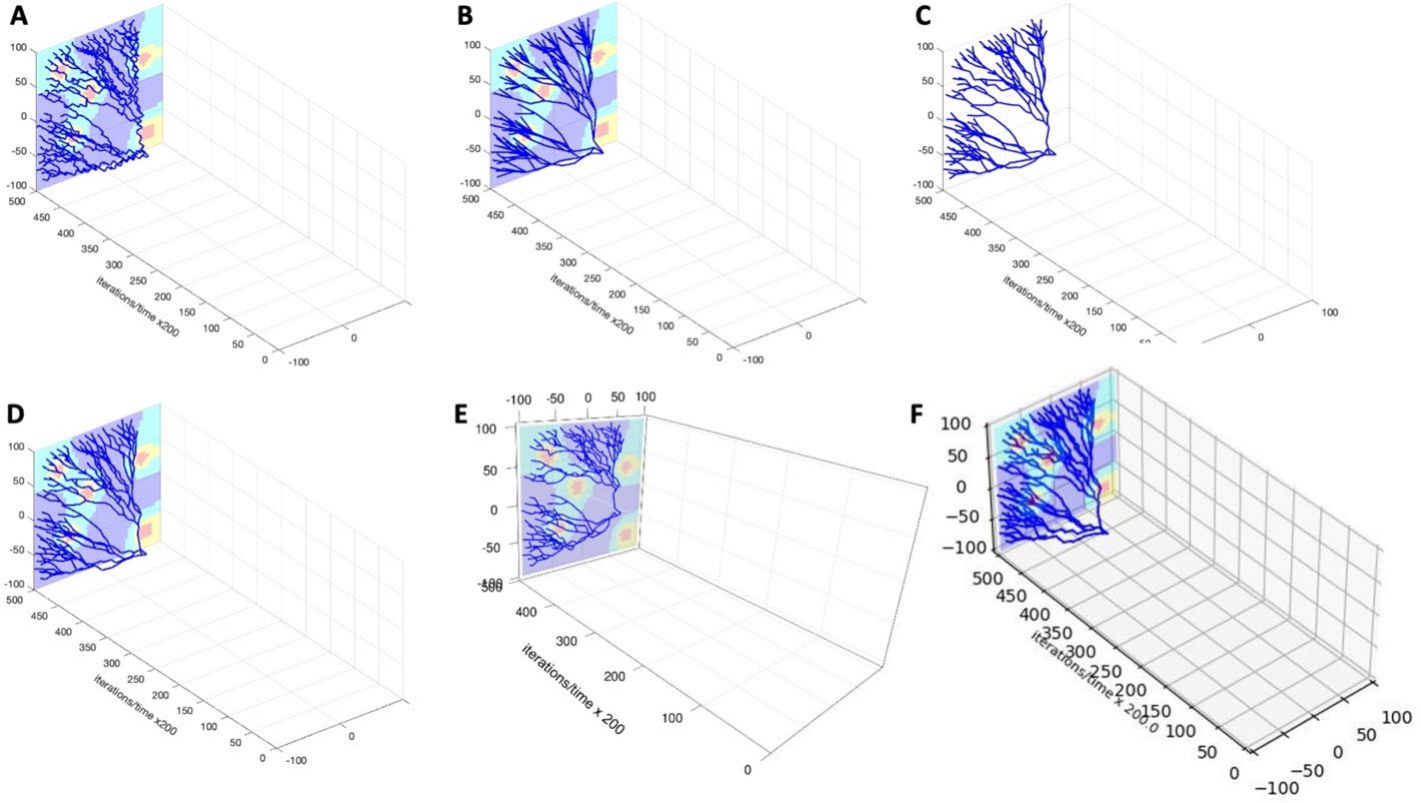
User-specified parameters and computational language options. **A** A 3D lineage tree of surviving cells for one clone (*LinG3DAliveClone*) drawn with data sampling parameter *fileStep* = 250; **B** The same lineage tree drawn with data sampling parameter *fileStep* = 5000; **C** The same lineage tree drawn with no background *IsGradient* = 0 and with data sampling parameter *fileStep* = 2500. **D**–**F** 3D lineage trees of the same single clone of surviving cells drawn with routines implemented in MATLAB (**D**), R (**E**), and Python (**F**), respectively (*fileStep* = 2000)

Each ***LinG3D*** routine requires that values of several input function arguments are specified by the user to represent their own simulation setup:

1. *pathData*—a text argument that defines the name of the main directory in which the subdirectory*/data/* must be located with all input data files.
2. *numClones or cloneNum*—a natural number argument that specifies either the total number of clones to be drawn (*numClones* in routines: ***LinG3DAll*** and ***LinG3DAliveAll***), or a specific clone number that will be drawn (*cloneNum* in routines: ***LinG3DClone*** and ***LinG3DAliveClone***); the clones are numbered starting at 0.
3. *xmin*, *xmax*, *ymin*, and *ymax*—four real number arguments that specify the domain boundaries $\left[{x}_{min},{x}_{max}\right]\times \left[{y}_{min},{y}_{max}\right]$ used to define a rectangular patch of a tissue.
4. *tmin* and *tmax*—two real number arguments that specify the initial and final iteration numbers. Note, that these numbers are used to optimize the spacing along the y-axis that represents the number of recorded data (*timeStep* variable). This is done for visualization purposes, such that all three axes have comparable scale.
5. *fileStep*—a natural number argument that specifies the progression step indicating frequency of the data to be sampled for the 3D lineage three visualization. Note that, more frequent data sampling will show more details of the simulation: smaller changes in cell locations between time points at which the branches are drawn, that results in more tortuous tree branches (Fig. 3A). On the other hand, less frequent data sampling for drawing will result in more linear tree branches since some intermediate cell positions will be omitted (Fig. 3B).
6. *IsGradient*—a binary number argument that indicates whether to draw the background concentration of a specific environmental factor or not: 1 to draw (Fig. 3B), 0 not to draw (Fig. 3C). The spatial distribution of the microenvironmental factor, such as oxygen or drug, is projected on the xz-plane. Note, that the provided concentration values (min and max drug levels) are used to optimize the split of concentration values into four groups, in order to draw each group in a different color (from the highest concentration to lowest: red—yellow—cyan—blue). This is done for visualization purposes and allows to use four distinct colors to draw the background gradient.
7. *toPrint*—a binary number argument that indicates whether to save the final figure or not (1 to save, 0 not to save).

This implementation is unified across the MATLAB, Python, and R routines.

#### Available routine versions

The *LinG3D* routines have been implemented in different computational systems and each of the discussed routines is provided in three computational languages: MATLAB (Fig. 3D), R (Fig. 3E), and Python (Fig. 3F). The following additional libraries are required to run the routines in R: *rgl* v1.3.1 for 3D visualization [8], *rapportools* v1.1 for vector and matrix operations [9], *readr* v2.1.2 for reading external files [10], and *devtools* v2.4.5 for GitHub installation [11]; MATLAB and Python routines do not require installation of any non-standard toolboxes or libraries.

## Results

To illustrate the results produced by each routine in the *LinG3D* package, we applied them to two simulated tumors that differ in the number of generated clones and one experimental colony growth assay with previously identified cellular clones. In both simulated cases, a growing colony of tumor cells is exposed to a cytotoxic drug, resulting in massive cell death. However, the acquired mutations make cells drug-resistant and allow for the emerging cellular clones to persist. These two examples vary only in one parameter: the probability of cell mutation which results in very different numbers of the generated cellular clones. In the experimental colony growth assay, the cells are grown in a drug-free medium in a standard well, where they can freely move and proliferate. Over the course of the experiment, the cells were able to divide a small number of times leading to the emergence of several small clones.

### Case study of a simulated tumor with a small number of clones

**Fig. 4:**
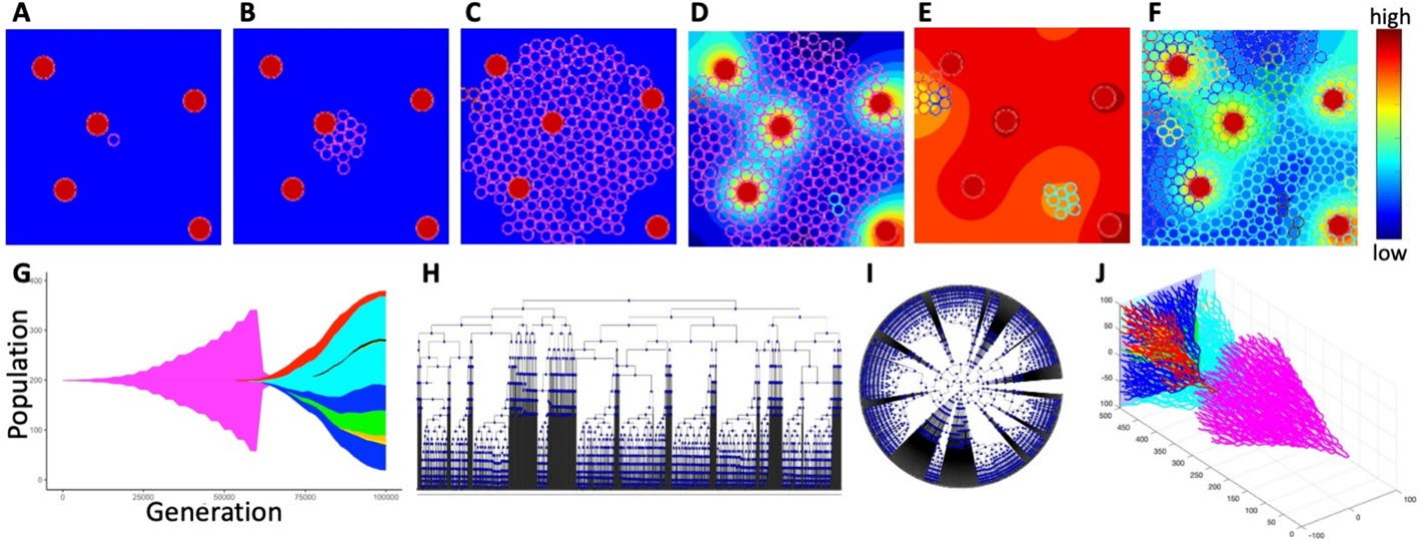
Evolution of a 9-clone tumor. Tumor growth simulated using *tumorGrowth_example1* with a parameter ${\mathcal{P}}^{mut}$=0.005: **A** an initial tumor cell (pink, iteration 0); **B** a growing cluster of non-mutated tumor cells (pink, iteration 18,000); **C** the emergence of the first mutated cell (red, top left edge, iteration 56,500); **D** the beginning of drug injection (second mutated clone in cyan, iteration 60,250); **E** drug-induced cell death (iteration 63,250); **F** the final configuration with 9 cellular clones (iteration 100,000). The color bar represents the drug concentration. **G** the corresponding tumor jellyfish evolution graph (*ggmuller* routine [2]); Clone colors correspond to cell colors in (**A**–**F**). **H**, **I** a classical lineage tree showing the relationship between mother and daughter cells in square and radial configuration (*phytree* routine [5]); **J **the corresponding full 3D lineage tree (our *LinG3DAll* routine)

In this computational model, a small patch of the tissue with five nonuniformly spaced vessels is used. A single tumor cell starts proliferating until a cell colony of a noticeable size is established. Then, the intravenously injected drug diffuses out of the vessels forming an irregular gradient within the tissue. All cells absorb the drug from their vicinity and can die if the accumulated drug level is high. Upon cell division, the daughter cells can mutate and the mutated cells acquire drug resistance giving rise to a mutated drug-resistant cellular clone. In this example, the probability of cell mutation is low (${\mathcal{P}}^{mut}$=0.005), thus, a small number (nine) of cellular clones is generated. In Fig. 4, six snapshots from this simulation are shown. Starting from the original mother cell (Fig. 4A), a small clone of non-mutated cells has developed (Fig. 4B, C). Since the probability of mutation is small, only two mutated cells emerged before the drug was injected (Fig. 4D). Extensive cell death is observed upon drug administration and its diffusion throughout the tissue (Fig. 4E). The surviving cells have now more space to proliferate as the overcrowding is no longer an issue. However, the number of clones remains small (9 at the end of the simulation) since the mutation probability is low. All mutated cells are drug-resistant which leads to a repopulation of the whole tissue (Fig. 4F). The details of this mathematical model are provided in the Supplement S4. The corresponding tumor jellyfish evolution graph (Fig. 4G) shows changes in clonal abundance (population) over time (generation). The color of each clone corresponds to the colors of cells in Fig. 4A–F. The classical phylogenetic tree, in which tree branches connect nodes representing a mother and their two daughter cells, and with the branch length corresponding to the evolution time, is shown in Fig. 4H in the horizontal configuration and in Fig. 4I in the radial configuration. The full 3D lineage tree is shown in Fig. 4J.

**Fig. 5:**
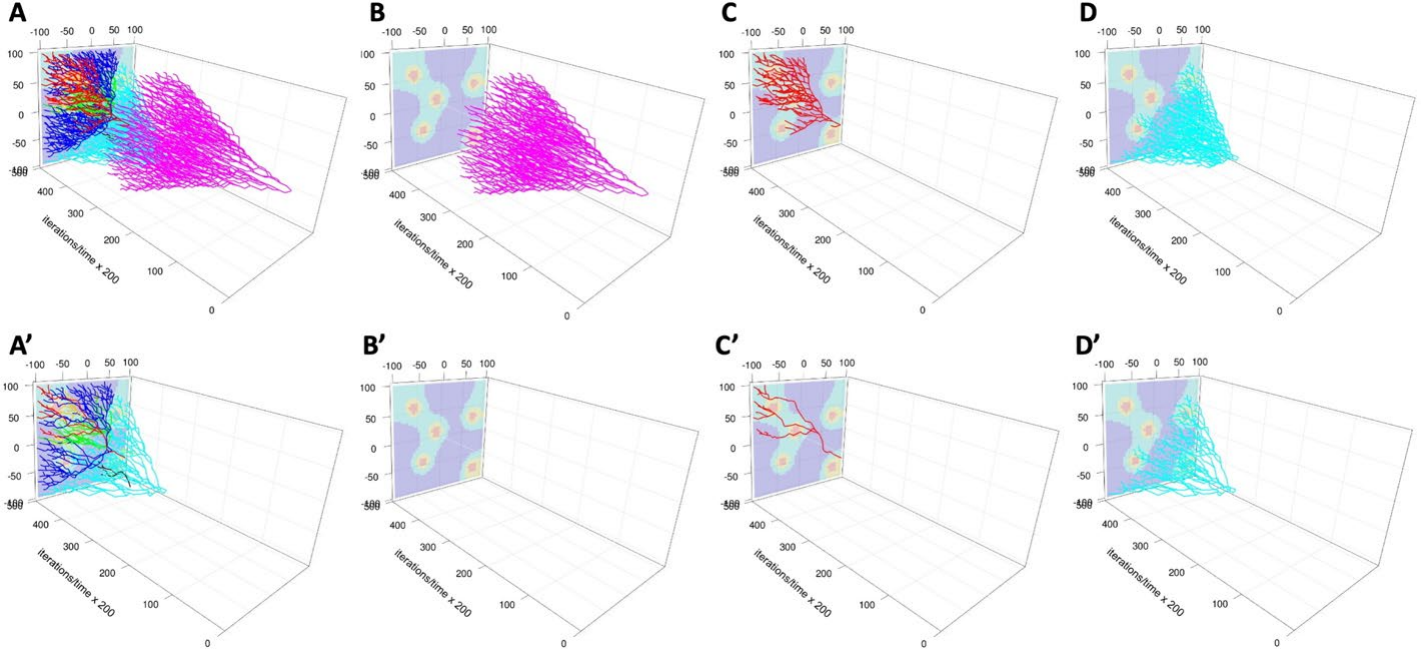
3D lineage trees of individual clones drawn with R routines. For each clone (denoted by a different color), the top row shows the 3D lineage tree with all cells belonging to that clone (**A** *LinG3DAll* and **B**–**D** *LinG3DClone* routines), while the bottom row includes only the cells that survived to the end of the simulation (**A’** *LinG3DAliveAll* and **B’**–**D’** *LinG3DAliveClone* routines). **B**–**B’** initial clone #0; **C**–**C’** mutated clone #1; **D**–**D’** mutated clone #2

To obtain a better understanding of the tumor spatio-temporal evolution, we inspected the 3D lineage trees of all clones together and of the three most prominent individual clones (out of 9) from this simulation, including the initial clone. Figure 5 (drawn with R-based routines) shows pairs of 3D lineage trees: trees that include traces of all cells belonging to a given clone (Fig. 5A–D) and trees that include only traces of cells that survived to the end of the simulation (Fig. 5A’–D’). Each clone is denoted by a different color. In all cases, the trees with alive cells are smaller than the trees with all cells, since many cells have died (due to random cell death or drug-induced cell death) or have left the computational domain. In some cases, there are no surviving cells—the most obvious example is the initial clone of drug-sensitive cells (clone #0, Fig. 5B’) for which all cells have died after the drug was administered. Other 3D lineage trees from this example drawn with MATLAB, Python, and R routines are included in Supplement S1.

To generate these 3D lineage trees, we had to ‘specify the following function arguments: data directory (pathData = ‘exampleB005’), spatial domain (xmin = −100 xmax = 100 ymin = − 100 ymax = 100), temporal domain (tmin = 0 tmax = 10,000), frequency of sampled data (fileStep = 250), the background indicator (IsGradient = 1), and the figure saving indicator (toPrint = 1). For drawing all clones, the total number of clones (numClones = 9) was specified. For drawing individual clones, the clone numbers (cloneNum = 0,1,2) were used, respectively. The presented 3D lineage trees show that the initial clone develops and spreads uniformly in space before the drug is injected (Fig. 5B) and then, upon drug diffusion, all these drug-sensitive cells die (Fig. 5B’). However, the two presented drug-resistant clones are more localized and grow to occupy specific niches: the top-left corner of the tissue (Fig. 5C) and the bottom-right corner (Fig. 5D). This information is not available from classical jellyfish evolution graphs (Fig. 4G) or phylogenic trees (Fig. 4H, J).

### Case study of a simulated tumor with a large number of clones

**Fig. 6:**
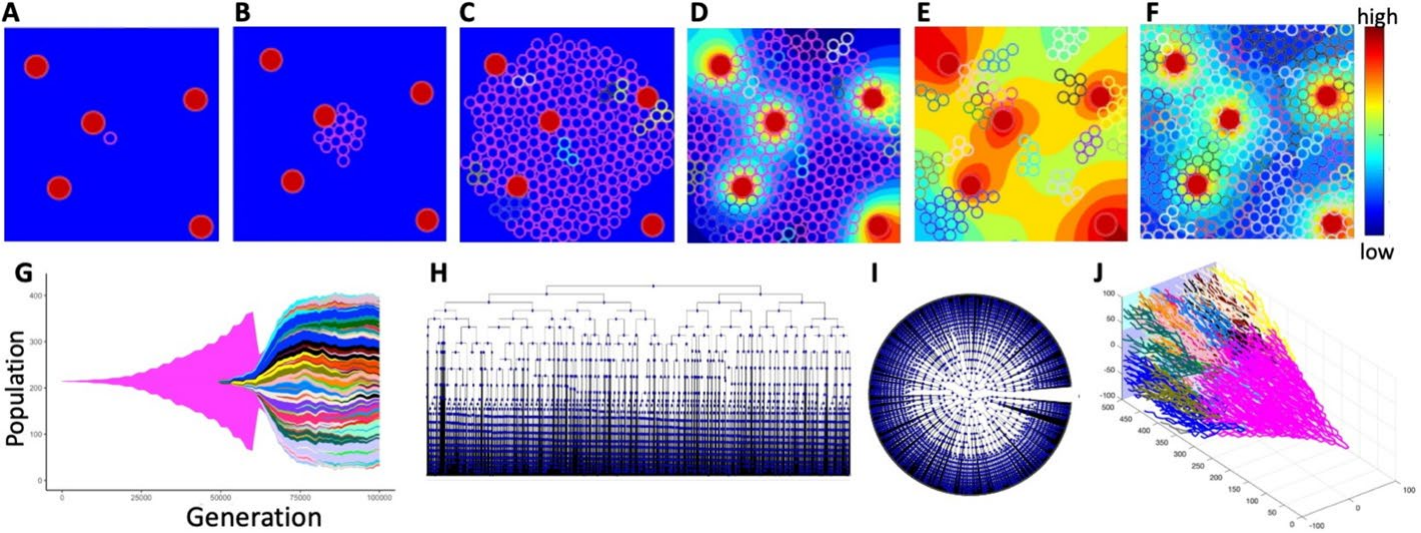
Evolution of a 147-clone tumor. Tumor growth simulated using *tumorGrowth_example2* with a parameter ${\mathcal{P}}^{mut}$=0.05: **A** an initial tumor cell (pink, iteration 0); **B** a growing cluster of non-mutated tumor cells (pink, iteration 18,000); **C** ten mutated cells have already emerged (multiple colors, iteration 56,500); **D** the beginning of drug injection (iteration 60,250); **E** drug-induced cell death (iteration 63,250); **F** the final configuration with 147 cellular clones (iteration 100,000); the color bar represents the drug concentration. **G** the corresponding tumor evolution graph (*ggmuller* routine [2]); clone colors correspond to cell colors in (**A**–**F**). **H**, **I** classical lineage trees showing the relationship between mother and daughter cells in square and radial configurations (*phytree* routine [5]). **J** the corresponding full 3D lineage tree (our *LinG3DAll* routine)

In the second example, the probability of cell mutation is higher (${\mathcal{P}}^{mut}$=0.05) resulting in a large number (147) of cellular clones that are also more dispersed within the tumor tissue when compared to the previous example. In Fig. 6, six snapshots from this simulation are shown. Like in the previous case, we start with one original mother cell (Fig. 6A) that generates a small clone of non-mutated cells (Fig. 6B). The growing tumor cell cluster is similar in shape to that in the previous example because we fixed the seed of the random number generator. However, since the probability of mutation is larger here, fifteen cells have mutated before the drug was administered (Fig. 6C, D). Drug injection and its intratumor diffusion lead again to a massive cell death (Fig. 6E) that allows more cells to proliferate as the cells are no longer overcrowded. More cell divisions also lead to an increase in the number of cell mutations (147 at the end of the simulation). All mutated cells are drug-resistant which leads to a repopulation of the whole tissue (Fig. 6F). The corresponding tumor evolution graph (Fig. 6G) shows changes in clonal abundance (population) over time (generation). The color of each clone corresponds to the colors of cells in Fig. 6A–E. The classical phylogenetic tree in the horizontal configuration is shown in Fig. 6H and in the radial configuration in Fig. 6I. The full 3D lineage tree is shown in Fig. 6J.

**Fig. 7:**
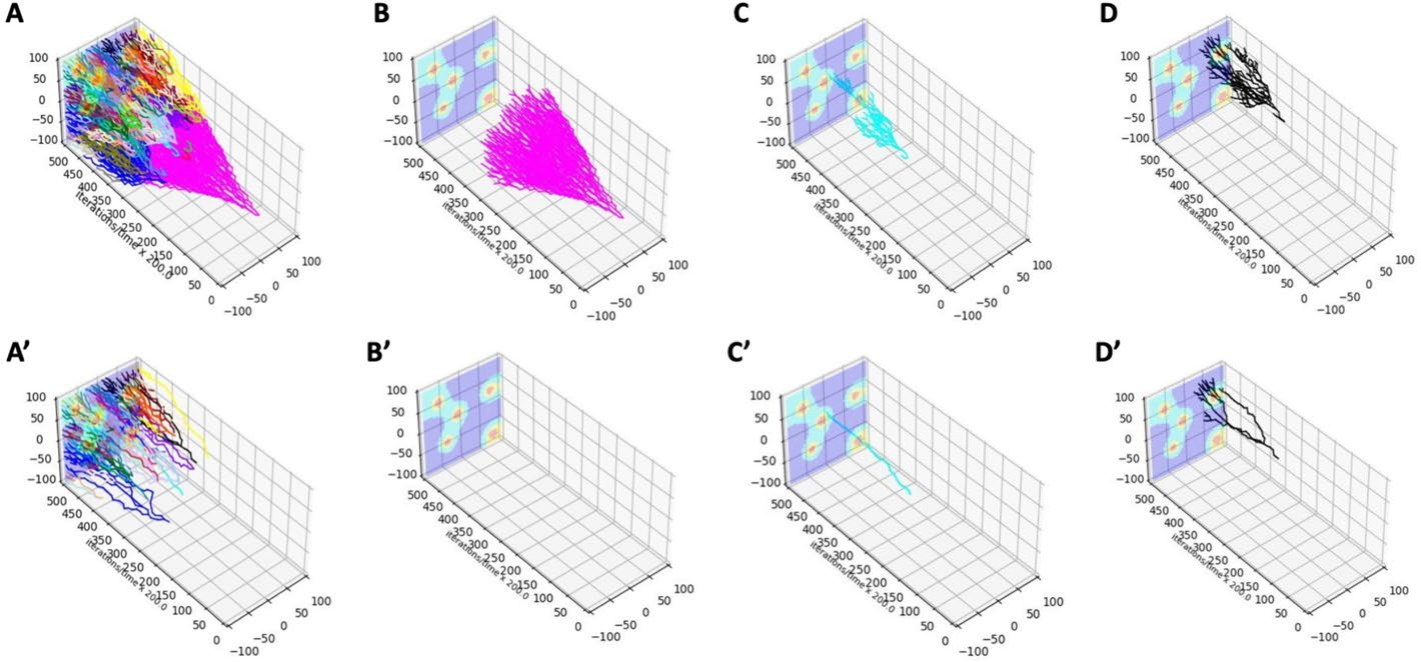
3D lineage trees of individual clones drawn with Python routines. For each clone (denoted by a different color), the top row shows the 3D lineage tree with all cells belonging to that clone (**A** *LinG3DAll* and **B**–**D** *LinG3DClone* routine), while the bottom row includes only the cells that survived to the end of the simulation (**A’** *LinG3DAliveAll* and **B’**–**D’** *LinG3DAliveClone* routine). **B**–**B’** initial clone #0; **C**–**C’** mutated clone #2; **D**–**D’** mutated clone #5

Since this simulation generated a large number of clones (147), we present here the 3D lineage trees for the three most prominent clones only. More graphs for other cellular clones (in MATLAB, Python, and R) are included in Supplement S2. Figure 7 (drawn with Python-based routines) shows pairs of 3D lineage trees with the top row including traces of all cells belonging to a given clone (Fig. 7A–D) and the bottom row including traces of cells that survived to the end of the simulation (Fig. 7A’–D’). As in the previous case, each clone is denoted by a different color. These clones are smaller and much less dispersed than in Example 1 since the cells have mutated more often and generated more clones. Again, the trees with alive cells are smaller than the trees with all cells, because many cells have died (due to random cell death or drug-induced cell death) or have left the computational domain. In some cases, there are no surviving cells, including the initial drug-sensitive clone (Fig. 7B’).

The two presented 3D lineage trees for drug-resistant clones (Fig. 7C, D) show how the cells move through the tissue to avoid the areas next to the vessels that contain very high drug concentrations. The surviving cells are localized in the low-drug (blue) regions (Fig. 7C’, D’). This information cannot be obtained from classical jellyfish evolution graphs (Fig. 6G) or phylogenic trees (Fig. 6H, J).

To generate these 3D lineage trees, we specified the following function arguments: data directory (pathData = ‘exampleB05’), spatial domain (xmin = −100 xmax = 100 ymin = − 100 ymax = 100), temporal domain (tmin = 0 tmax = 10,000), frequency of sampled data (fileStep = 250), the background indicator (IsGradient = 1), and the figure saving indicator (toPrint = 1). For drawing all clones, the total number of clones (numClones = 147) was specified. For drawing individual clones, the clone numbers (cloneNum = 0,2,5) were used, respectively.

### Case study of an experimental colony growth assay with identified cellular clones

**Fig. 8:**
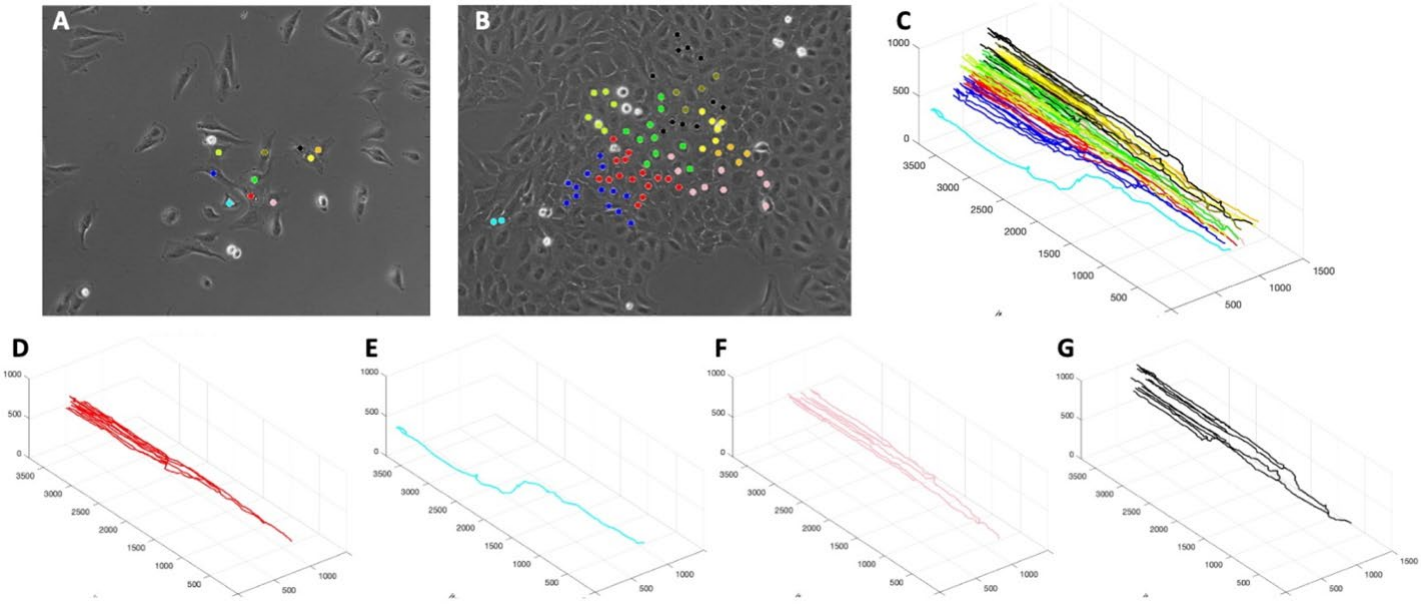
3D lineage trees (MATLAB routines) for individual clones identified in an experimental assay. **A**, **B** the initial and final frames from a bright field time-lapse microscopy of a growing cell colony with 10 different clones indicated by different colors. **C** 3D lineage trees for all ten cellular clones (*LinG3DAll*); **D**–**G** 3D lineage trees for all cells in four selected cellular clones (*LinG3DClone*); each tree color corresponds to cells’ colors in (**A** and **B**)

While our routines were developed to visualize data from computational model simulations, they can also be applied to experimental data, provided that data is recorded in the required format. As an illustration, we will visualize clones generated during the 2D growth assay where a sequence of images recorded 12 min apart was annotated using in-house MATLAB code to identify the coordinates of cells arising from 10 precursor cells chosen from an initial image. The mother cell-daughter cell relation was also recorded whenever one of the traced cells had divided. The initial image with ten selected cells is shown in Fig. 8A, and the final image with cells forming the ten clones is shown in Fig. 8B (additional images are included in Supplement S3). Each clone is drawn with a different color. The full 3D lineage tree of all ten cellular clones (drawn with *LinG3DAll*, MATLAB-based version), is shown in Fig. 8C, and four specific 3D lineage trees for individual cellular clones (drawn with *LinG3DClone*, MATLAB-based version) are shown in Fig. 8D–G.

To generate these 3D lineage trees, we had to specify the following input function arguments: data directory (pathData = ‘exampleExp’), spatial domain (xmin = 0 xmax = 1500 ymin = 0 ymax = 1000), temporal domain (tmin = 0 tmax = 864), frequency of sampled data (fileStep = 4), the no background indicator (IsGradient = 0), and the figure saving indicator (toPrint = 1). For drawing all clones, the total number of clones (numClones = 10) was specified. For drawing individual clones, the clone numbers (cloneNum = 1,2,10,5) was used, respectively.

The presented spatio-temporal lineage trees for individual cells revealed that the cell in Fig. 8E was quite motile and did not proliferate often, in contrast to other cells (Fig. 8D, F, G) that were dividing several times but did not move out at significant distances. These spatial effects are not observed in the classical phylogenic trees or jellyfish evolution graphs.

In the presented example, the U2OS (ATCC HTB-96) sarcoma cell line obtained from ATCC (Manassas, VA) was grown in a 2D monolayer in Dulbecco’s modified Eagle’s medium supplemented with 10% fetal bovine serum, 1% (v/v) penicillin–streptomycin, and 1% (v/v) L-glutamine at 37uC in a 5% CO_2_ incubator. Leica confocal microscope was used to record images of the same part of the well every 3 min for 96 h. However, the particular cell line used in this experiment as well as experimental conditions are not crucial for applying our routines. As long as cell history and cell locations can be identified in the longitudinal way, the *LinG3D* routines can be applied to other cell lines and other experimental 2D assays.

## Conclusions

The proposed by us *LinG3D* routines generate three-dimensional spatio-temporal trees that show the emergence of cellular clones in time, like classical lineage trees, as well as in space within a patch of the tumor tissue. These routines were designed primarily for application to data generated from simulations of tumor evolution models. However, they can also be applied to experimental cellular clones, if appropriate information can be collected. The routines can illustrate clonal development in growing tumors with or without exposure to anti-cancer treatments. In particular, they can enable analysis of spatial intra-tumor heterogeneity, such as the spatial distribution of cells with specific phenotypes or mutations.

We have previously used the 3D lineage tree routines to inspect tumor areas where drug-induced resistance can arise. These methods allowed us to identify three distinct microenvironmental niches that are prone to the emergence of clones with acquired drug resistance: sanctuaries with low drug exposure, protectorates with medium drug exposure but dense cellularity, and hypoxia niches with low cell proliferation rates [12, 13]. Such biological insights would not be recognized without the use of 3D cell lineage trees.

We have shown here that our routines are versatile with respect to the type of data (synthetic or experimental), number of visualized clones (from tens to hundreds), number of visualized time points, domain sizes, and computational languages (three popular: R, Python, and MATLAB are included here). The most elaborated 3D lineage tree from our examples is shown in Fig. 7A; it took 7 min to draw this tree on an Apple laptop computer. While these routines can handle a larger number of clones, the typical 2D jellyfish evolution graphs are applied to cases with the number of clones in tens rather than hundreds, as in the cases we showed in this paper. Future extensions of these routines can include options for changing clone colors in a personalized way, choosing the resolution for the background image of microenvironmental factor, or saving the generated images in different formats. Additionally, an option to draw several selected individual clones in the same image could be added.

In summary, visualization of cellular clones in both time and space can provide additional information on whether the particular cells or clones are motile vs. those that are more stationary; whether growing cells spread widely or hover around specific tissue niches. This can help in formulating new research hypotheses.

## Availability and requirements

Project name: *LinG3D*, Project home page: https://github.com/rejniaklab/LinG3D, Operating system(s): Mac OS, Programming language: MATLAB, Python, and R, Other requirements: Python v.3.8 or higher, MATLAB 2020 or higher, R v.4.1.1 or higher, License: GNU General Public License v3.0, Any restrictions to use by non-academics: none.

### Supplementary Information

Supplementary Material 1.

## Data Availability

All software code and data are available from the following depository: https://github.com/rejniaklab/LinG3D, https://github.com/rejniaklab/pyLinG3D, https://github.com/rejniaklab/r_LinG3D

## Abbreviations

*LinG3D*: Lineage graph in 3D
*LinG3DAll*: Lineage graph in 3D all cells and clones
*LinG3DAliveAll*: Lineage graph 3D all alive cells
*LinG3DClone*: Lineage graph 3D one specified clone
*LinG3DAliveClone*: Lineage graph 3D all alive cells from one specified clone
