# Supplementary material for: LinG3D: visualizing the spatio-temporal dynamics of clonal evolution
Source: BMC Bioinformatics. 2024 May 27;25:201. doi: 10.1186/s12859-024-05813-7 (PMC11131251; doi:10.1186/s12859-024-05813-7)
Supplement: Supplementary file 1 — Supplementary Material 1. [file 12859_2024_5813_MOESM1_ESM.pdf]

LinG3D: Visualizing the Spatio-Temporal Dynamics of Clonal Evolution

Anjun Hu, Awino Maureiq E. Ojwang', Kayode D. Olumoyin, Katarzyna A. Rejniak

Supplemental Material S1.

Examples of 3D lineage trees in MATLAB, Python, and R from a simulation of a growing tumor with a small number of cellular clones (probability of mutation: $\mathcal{P}^{mut}$=0.005).

To obtain a better understanding of tumor spatio-temporal evolution, we inspected the 3D lineage trees of the six largest cellular clones (out of 9) from this simulation, including the initial clone. Fig.S1-S3 show pairs of 3D lineage trees using our *LinG3D* routines in MATLAB (Fig.S1), Python (Fig.S2), and R (Fig.S3). Top rows show trees that include traces of all cells belonging to a given clone (Fig.S1-S3A-F); bottom rows show trees that include only traces of cells that survived to the end of the simulation, that is without branches with dead cells (Fig.S1-S3A'-F'). Each clone is denoted by a different color. In all cases, the trees with alive cells are smaller than the trees with all cells, since many cells have died (due to random cell death or drug-induced cell death) or have left the computational domain. In some cases (Fig.S1-S3A') there are no surviving cells—the most obvious is the initial clone of drug-sensitive cells (clone #0) for which all cells have died after the drug was administered.

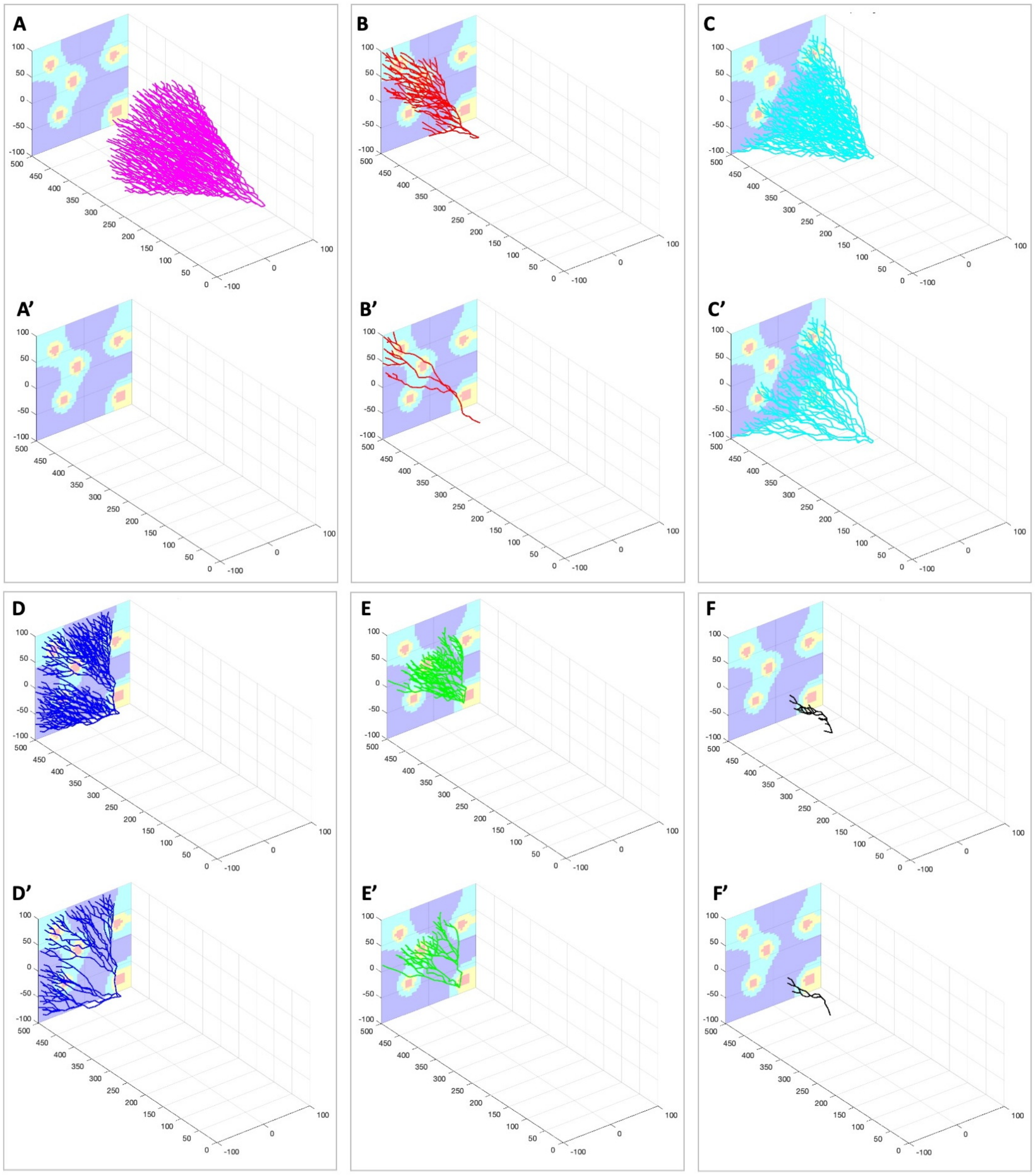

Fig.S1. 3D lineage trees of individual clones drawn with MATLAB routines. For each clone (denoted by a different color), the top row shows the 3D lineage tree with all cells belonging to that clone (*LinG3DClone.m* routine), while the bottom row includes only the cells that survived to the end of the simulation (*LinG3DAliveClone.m* routine). **A-A’:** initial clone #0; **B-B’:** mutated clone #1; **C-C’:** mutated clone #2; **D-D’:** mutated clone #3; **E-E’:** mutated clone #4; **F-F’:** mutated clone #5.

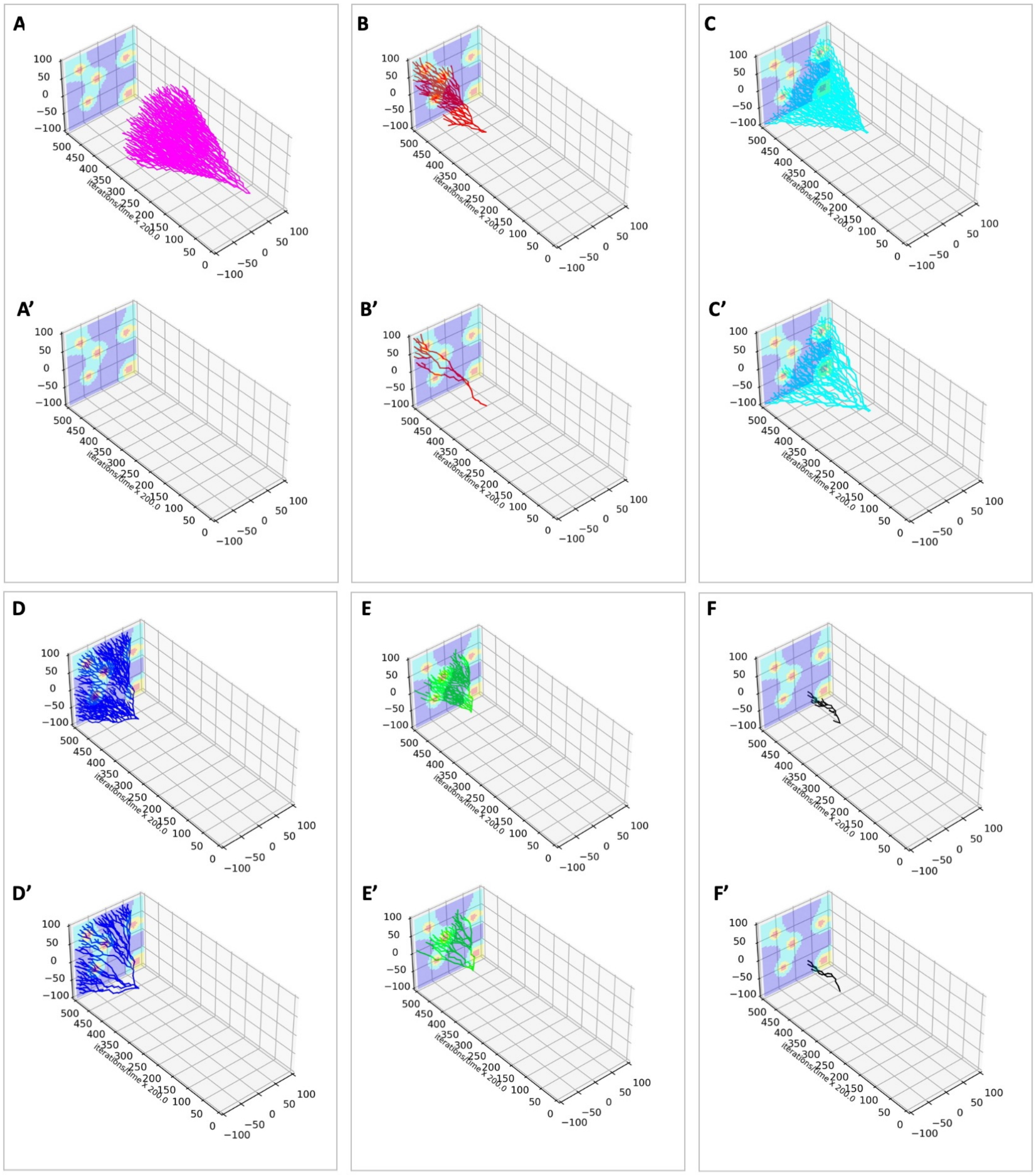

Fig.S2. 3D lineage trees of individual clones drawn with Python routines. For each clone (denoted by a different color), the top row shows the 3D lineage tree with all cells belonging to that clone (*LinG3DClone.py* routine), while the bottom row includes only the cells that survived to the end of the simulation (*LinG3DAliveClone.py* routine). **A-A’:** initial clone #0; **B-B’:** mutated clone #1; **C-C’:** mutated clone #2; **D-D’:** mutated clone #3; **E-E’:** mutated clone #4; **F-F’:** mutated clone #5.

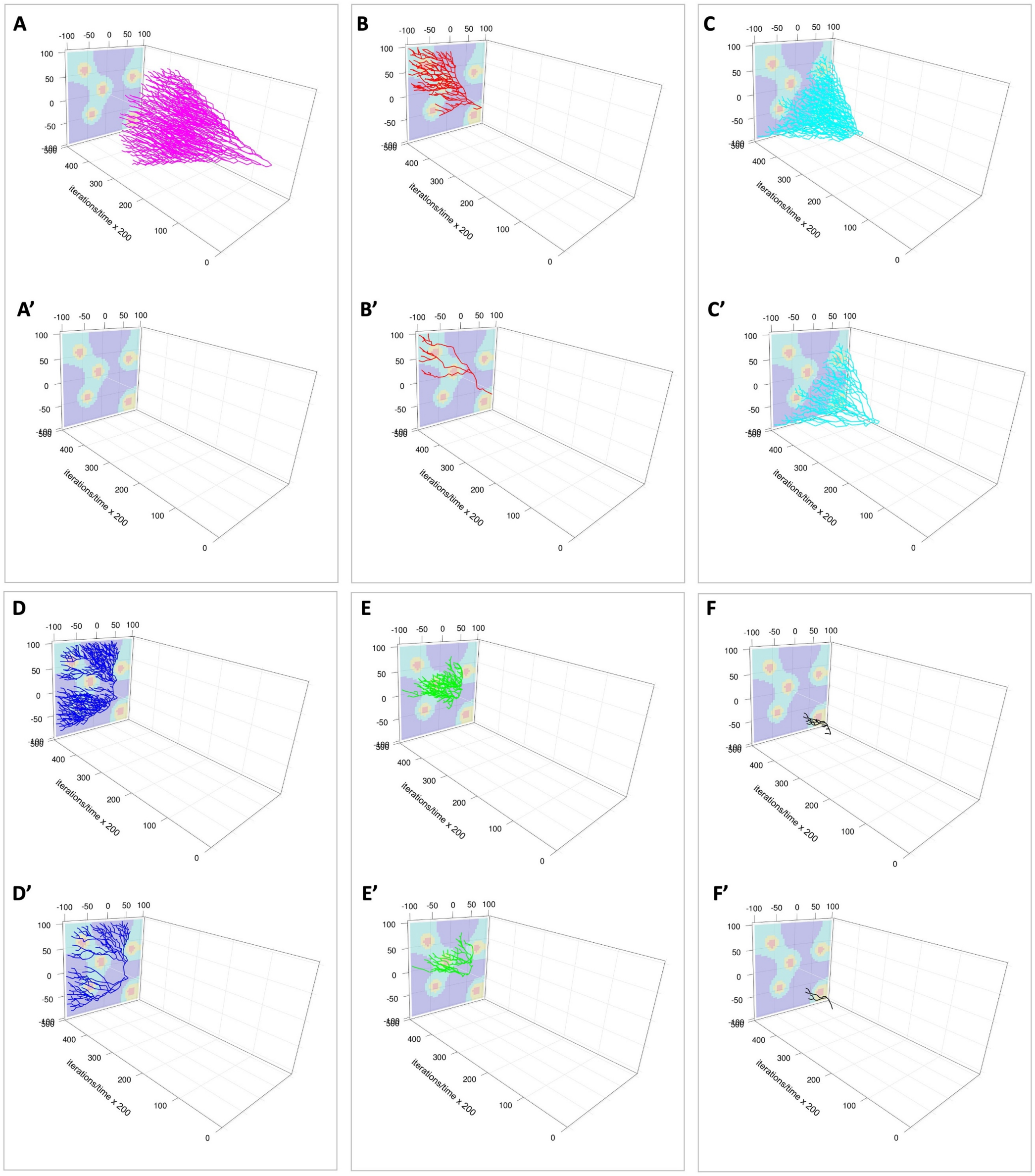

Fig.S3. 3D lineage trees of individual clones drawn with R routines. For each clone (denoted by a different color), the top row shows the 3D lineage tree with all cells belonging to that clone (*LinG3DClone.r* routine), while the bottom row includes only the cells that survived to the end of the simulation (*LinG3DAliveClone.r* routine). **A-A':** initial clone #0; **B-B':** mutated clone #1; **C-C':** mutated clone #2; **D-D':** mutated clone #3; **E-E':** mutated clone #4; **F-F':** mutated clone #5.

Supplemental Material S2.

Examples of 3D lineage trees in MATLAB, Python, and R from a simulation of a growing tumor with a large number of cellular clones (probability of mutation: $\mathcal{P}^{mut}$=0.05).

Since this simulation has generated a large number of clones (147), we focus here only on a small subset (12) of the largest clones and present their 3D lineage trees. Fig.S4-S6 show pairs of 3D lineage trees using our *LinG3D* routines in MATLAB (Fig.S4), Python (Fig.S5), and R (Fig.S6). Top rows show tress that include traces of all cells belonging to a given clone (Fig.S4-S6A-L); bottom rows show trees that include only traces of cells that survived to the end of the simulation (Fig.S4-S6A'-L'). As in the previous case, each clone is denoted by a different color. These clones are smaller and much less dispersed than in the previous example since the cells have mutated more often and generated more clones. Again, the trees with alive cells are smaller than the trees with all cells, since many cells have died (due to random cell death or drug-induced cell death) or have left the computational domain. In some cases, there are no surviving cells, including the initial drug-sensitive clone (Fig.S4-S6A'). The late clones, that is the clones emerging later during the tumor development, are smaller, and the majority of clones have not survived to the end of the simulation—more than 80 out of 147 (results not shown).

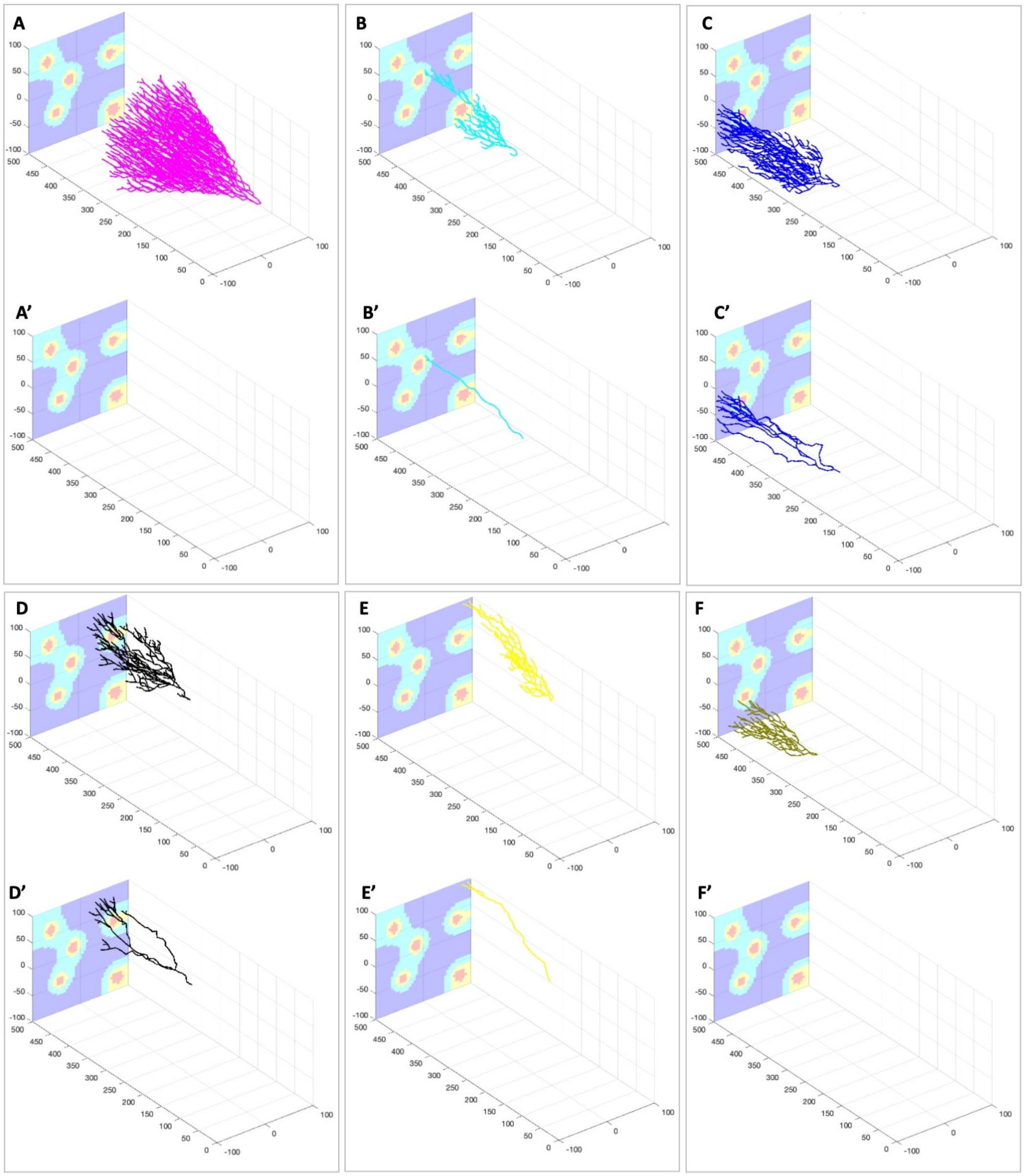

Fig.S4. 3D lineage trees of individual clones drawn with MATLAB routines. For each clone (with a different color) the top row includes the 3D lineage tree with all cells (*LinG3DClone.m* routine), while the bottom row includes only the cells that survived to the end of the simulation (*LinG3DAliveClone.m* routine). **A-A':** initial clone #0; **B-B':** mutated clone #2; **C-C':** mutated clone #3; **D-D':** mutated clone #5; **E-E':** mutated clone #7; **F-F':** mutated clone #9.

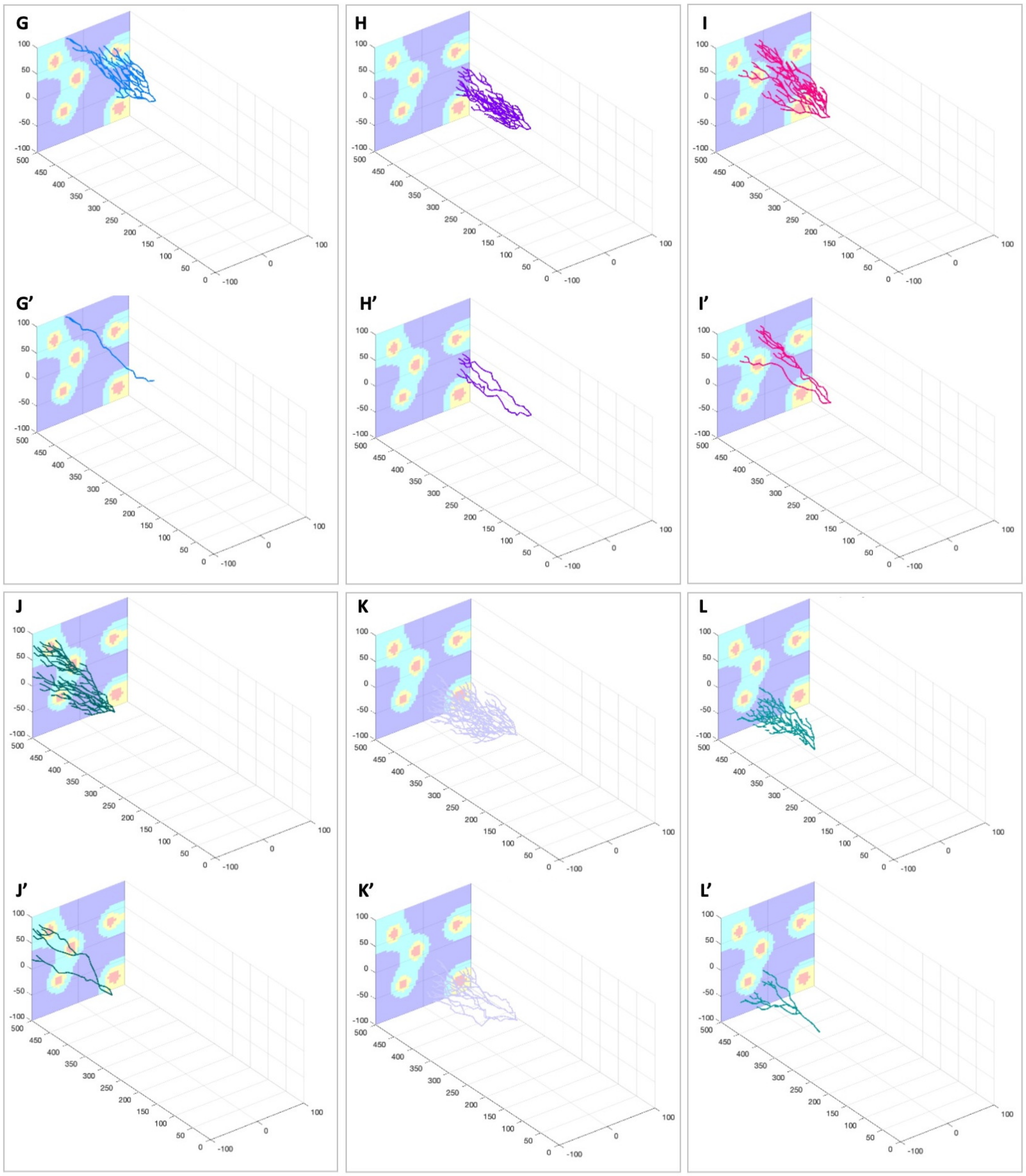

Fig.S4 (cont). 3D lineage trees of individual clones drawn with MATLAB routines. For each clone, the top row includes the 3D lineage trees with all cells (drawn with *LinG3DClone.m*), while the bottom row includes only the cells that survived to the end of the simulation (drawn with *LinG3DAliveClone.m*). **G-G’:** mutated clone #12; **H-H’:** mutated clone #14; **I-I’:** mutated clone #16; **J-J’:** mutated clone #19; **K-K’:** mutated clone #20; **L-L’:** mutated clone #25.

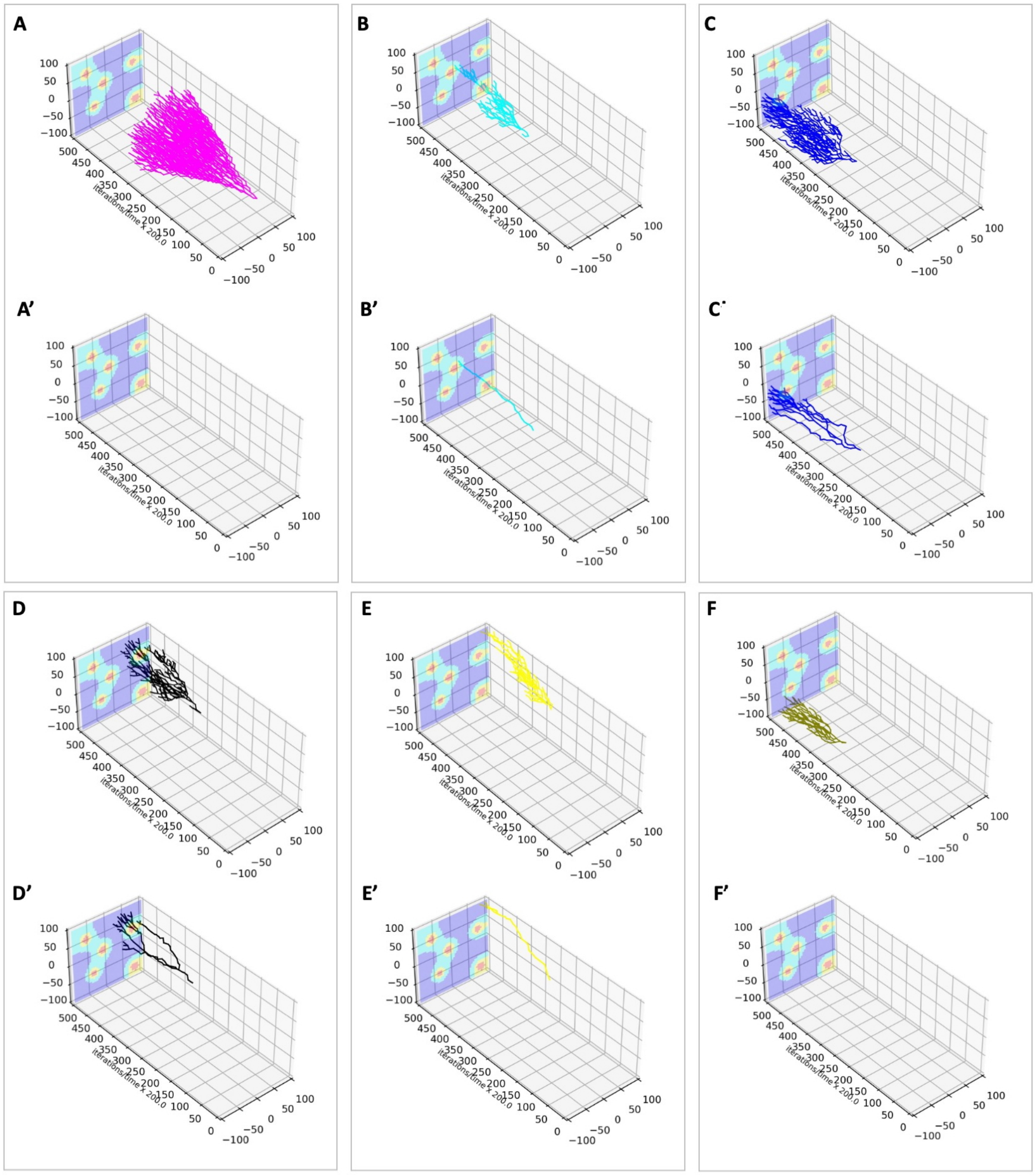

Fig.S5. 3D lineage trees of individual clones drawn with Python routines. For each clone (with a different color) the top row includes the 3D lineage tree with all cells (*LinG3DClone.py* routine), while the bottom row includes only the cells that survived to the end of the simulation (*LinG3DAliveClone.py* routine). **A-A’:** initial clone #0; **B-B’:** mutated clone #2; **C-C’:** mutated clone #3; **D-D’:** mutated clone #5; **E-E’:** mutated clone #7; **F-F’:** mutated clone #9.

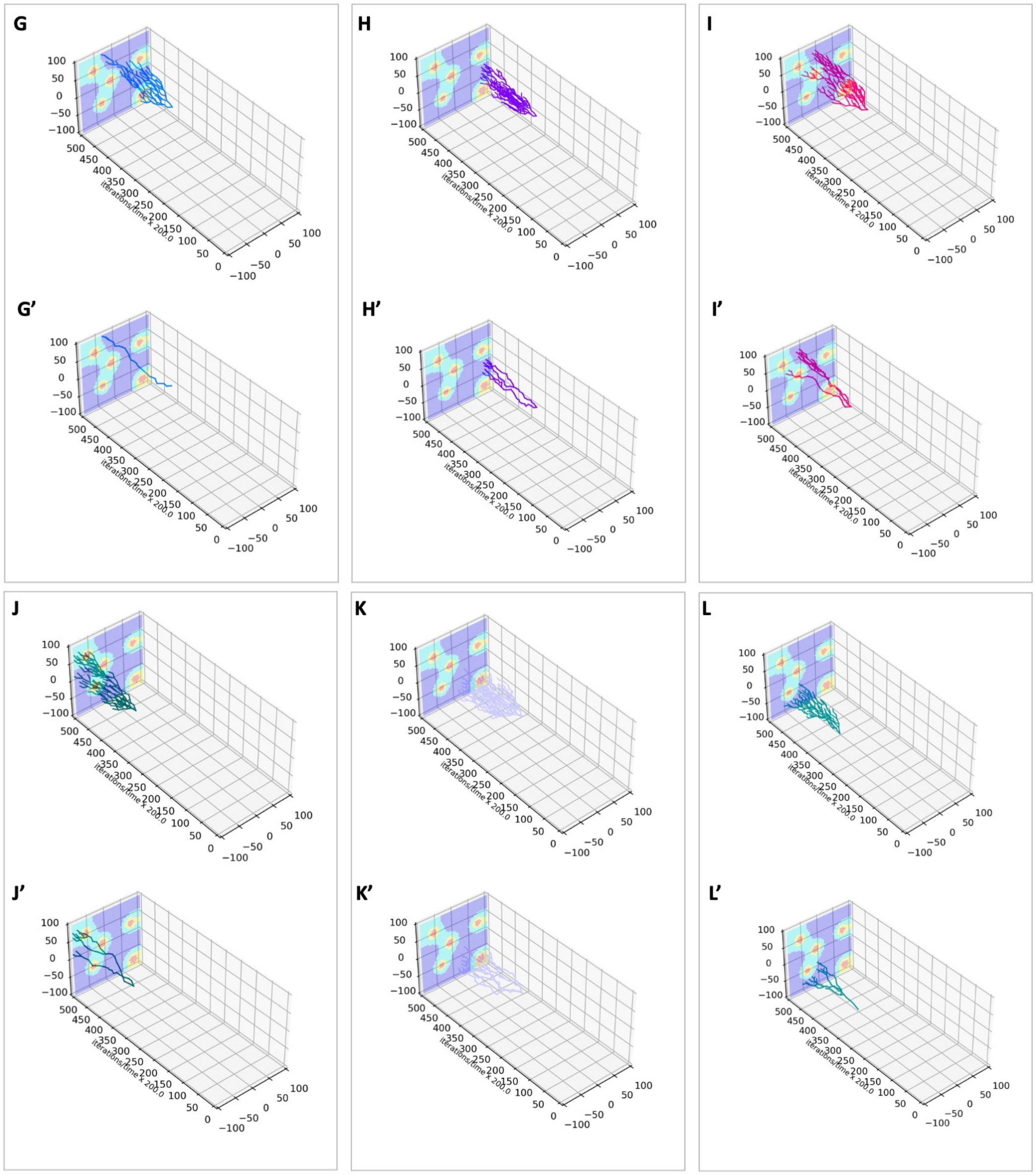

Fig.S5 (cont). 3D lineage trees of individual clones drawn with Python routines. For each clone, the top row includes the 3D lineage trees with all cells (drawn with *LinG3DClone.py*), while the bottom row includes only the cells that survived to the end of the simulation (drawn with *LinG3DAliveClone.py*). **G-G’:** mutated clone #12; **H-H’:** mutated clone #14; **I-I’:** mutated clone #16; **J-J’:** mutated clone #19; **K-K’:** mutated clone #20; **L-L’:** mutated clone #25.

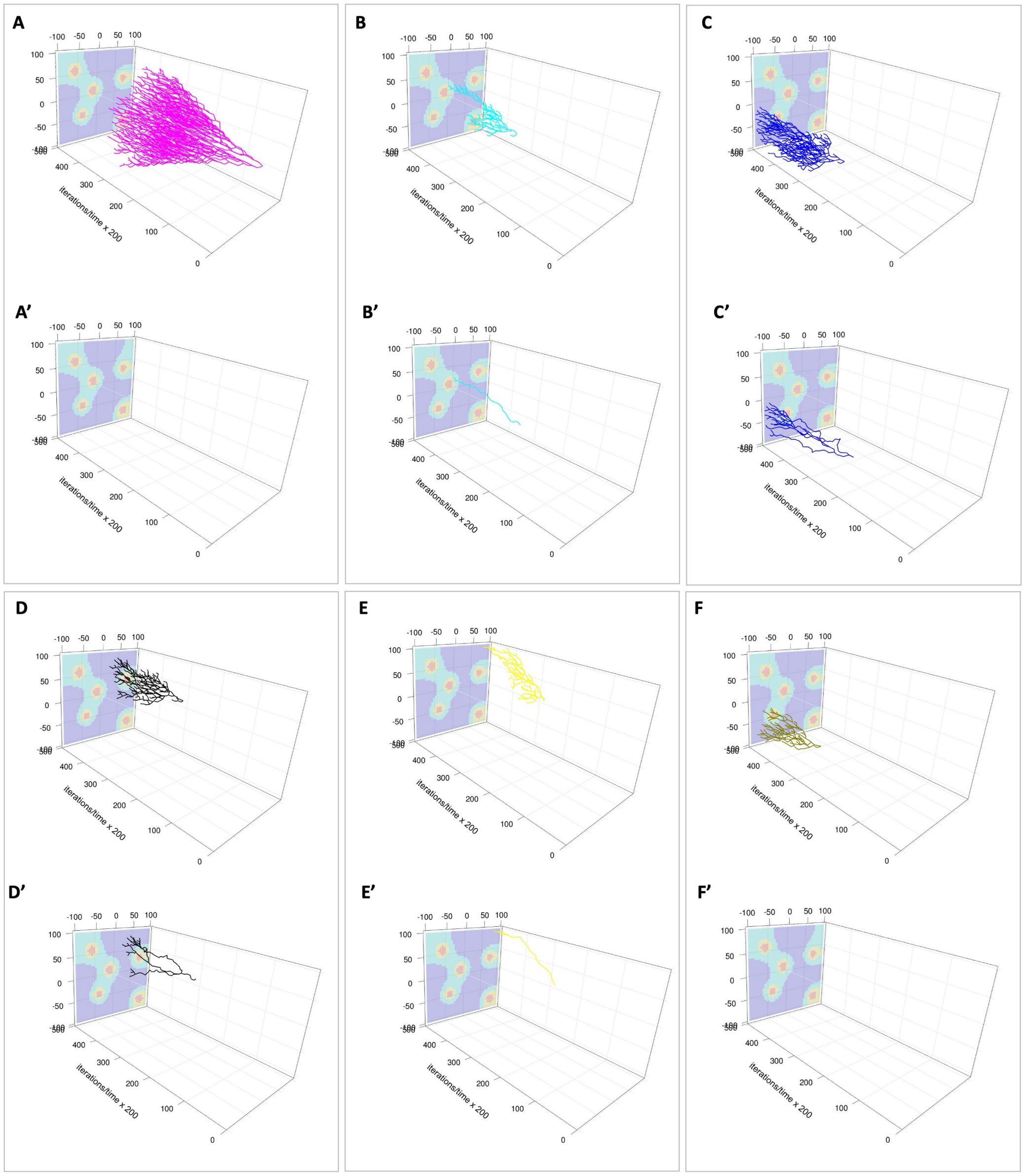

Fig.S6. 3D lineage trees of individual clones drawn with R routines. For each clone (with a different color) the top row includes the 3D lineage tree with all cells (*LinG3DClone.r* routine), while the bottom row includes only the cells that survived to the end of the simulation (*LinG3DAliveClone.r* routine). **A-A':** initial clone #0; **B-B':** mutated clone #2; **C-C':** mutated clone #3; **D-D':** mutated clone #5; **E-E':** mutated clone #7; **F-F':** mutated clone #9.

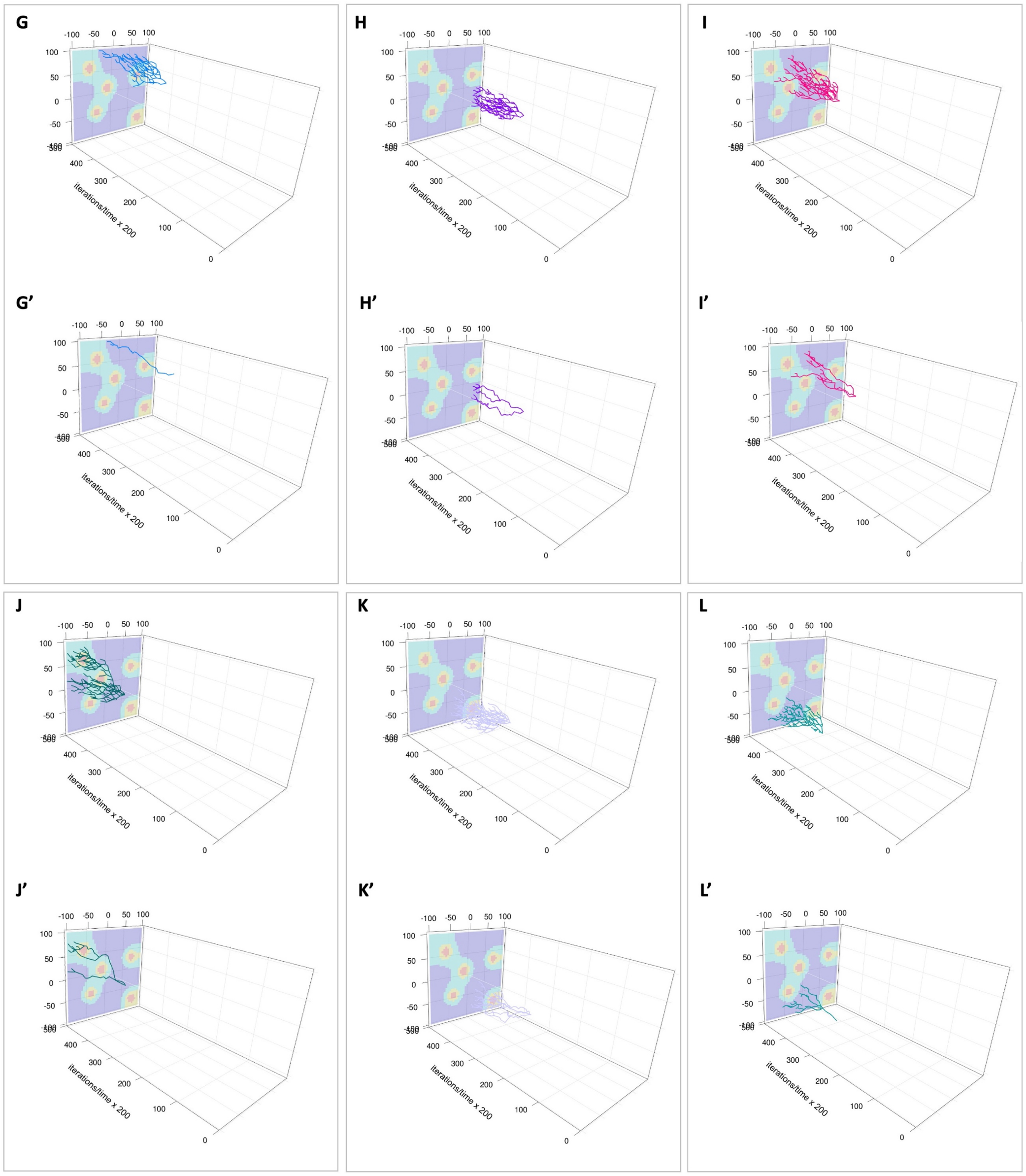

Fig.S6 (cont). 3D lineage trees of individual clones drawn with R routines. For each clone, the top row includes the 3D lineage trees with all cells (drawn with *LinG3DClone.r*), while the bottom row includes only the cells that survived to the end of the simulation (drawn with *LinG3DAliveClone.r*). **G-G':** mutated clone #12; **H-H':** mutated clone #14; **I-I':** mutated clone #16; **J-J':** mutated clone #19; **K-K':** mutated clone #20; **L-L':** mutated clone #25.

Supplemental Material S3.
Examples of cellular clones and 3D lineage trees from cell culture

To illustrate the use of *LinG3D* routines for experimental data, we traced ten cellular clones in the series of bright field images of the 2D culture of U2OS sarcoma cells. The images were acquired automatically every 3 minutes for 96 hours, and eight annotated images from this experiment are shown in Fig.S7A-H. For each annotated image, we recorded coordinates of all cells arising from the 10 selected precursor cells (shown in Fig.S7A) and coordinated of two daughter cells if the traced cell has divided. The full 3D lineage trees for nine clones are shown in Fig.S7I-R. Their colors correspond to the color of the selected cells.

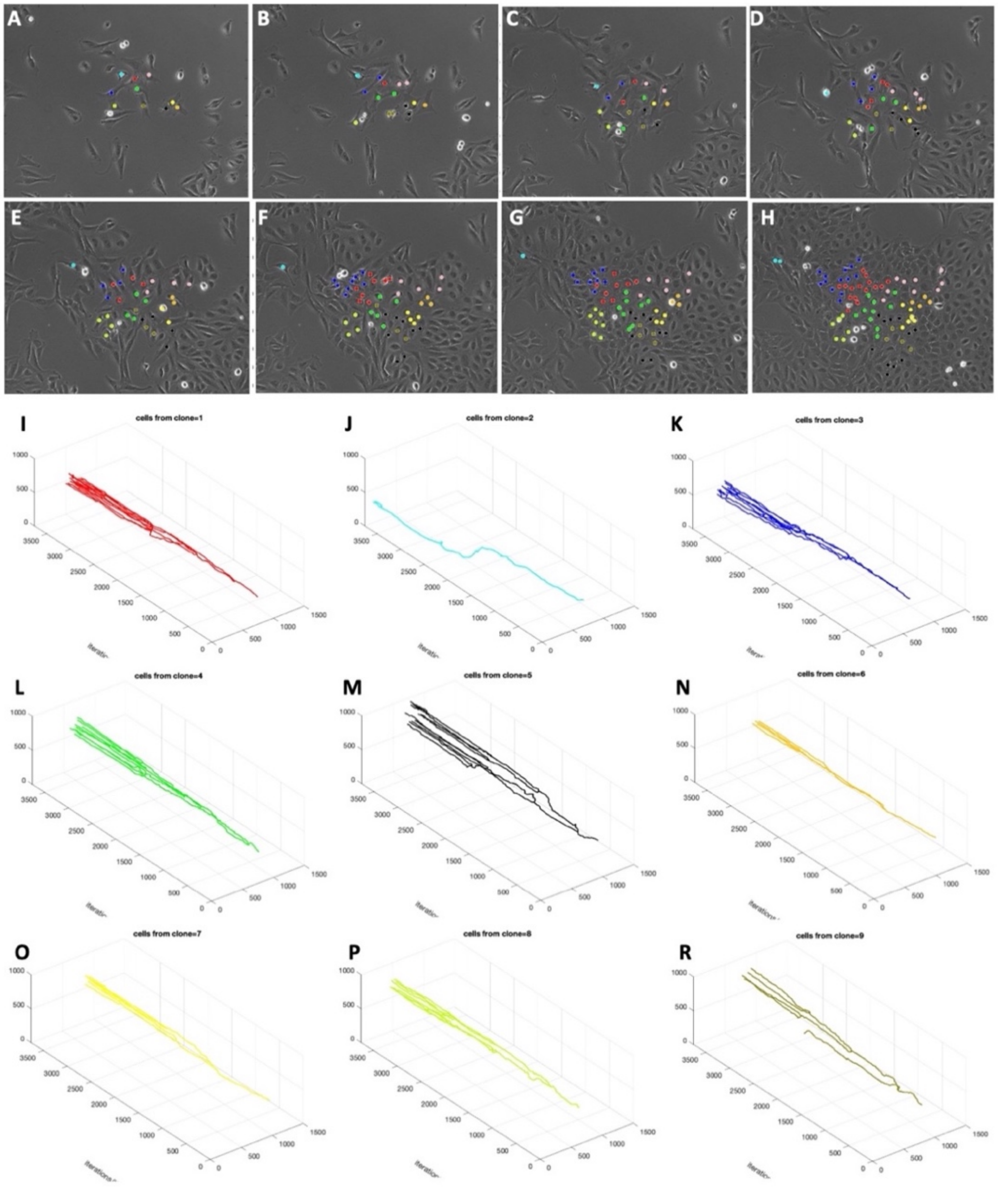

Fig.S7 Cellular clones and 3D lineage trees from in vitro culture. **A-H.** bright field images of in vitro culture of U2OS sarcoma cells with identified ten cellular clones. **I-R.** 3D lineage trees with all cells for 9 out of 10 cellular clones for the cell culture (drawn with *LinG3DClone.m*),

Supplemental Material S4.
Mathematical equations from a model for S1 and S2

To illustrate how the 3D lineage tree algorithms can be applied, we developed a relatively simple agent-based model of the growing tumor exposed to a cytotoxic drug (MATLAB routine: *tumorGrowth_example1.m* and *tumorGrowth_example2.m*). This model is based on our Multi-Cell Lattice-Free, *MultiCell-LF*, framework [1-3] in which individual cells interact with one another through physical forces and with the surrounding microenvironment via chemical factors, such as drugs or oxygen.

Computational domain
The model is defined on a square tissue patch: $[-100,100] \times [-100,100]\ \mu m^2$, that contains five irregularly distributed stationary vessels $\mathcal{V}_i$ $(i = 1, \dots, 5)$ that are the source of a cytotoxic drug $\gamma(x, y, t)$. The tumor cells X_i $(i = 1, \dots, \mathcal{N}_c)$ can proliferate, absorb the drug, undergo random and drug-induced death, and can mutate which results in cell resistance to the drug. The initial condition consists of a single tumor cell located in the middle of the domain with no drug. The no-flux boundary conditions are imposed on the drug domain. The cells are also removed from the system if they move outside of the domain boundaries.

Drug kinetics
Drug distribution within the tumor tissue depends on three factors: the amount of drug supplied by the vessels, the amount of drug taken up by the tumor cells, and the spatial localization of all blood vessels and cells. The drug $\gamma(x, y, t)$ is supplied from each vessel $\mathcal{V}_i$ with the influx rate $\mathcal{I}_\gamma$, it diffuses through the tissue with a constant diffusion coefficient $\mathcal{D}_\gamma$, and is absorbed by each cell X_i with the uptake rate $\mathcal{U}_\gamma$ proportional to the drug concentration $\gamma(x, y, t)$. The equation of drug kinetics is defined as follows:

$$\frac{\partial \gamma(x,y,t)}{\partial t} = \underbrace{\mathcal{I}_\gamma \cdot \sum_{i=1}^{\mathcal{N}_v} \chi_{\mathcal{R}_v}\big(\mathcal{V}_i, (x,y)\big)}_{influx\ supply} + \underbrace{\mathcal{D}_\gamma \Delta\gamma(x,y,t)}_{diffusion} - \underbrace{\mathcal{U}_\gamma \cdot \gamma(x,y,t) \cdot \sum_{i=1}^{\mathcal{N}_c} \chi_{\mathcal{R}_c}(X_i, (x,y))}_{Celluar\ uptake}$$

where interactions between the drug grid (x, y) and the individual cells or vessels ($Z = \mathcal{V}_i$ or X_i) are specified by the indicator function with radius $\mathcal{R}$:

$$\chi_{\mathcal{R}}((x,y), Z) = \begin{cases} 1 & if\ \|(x,y) - Z\| < \mathcal{R} \\ 0 & otherwise \end{cases}$$

Cell division and mutation
Each cell is defined by its position X_i and a constant radius $\mathcal{R}_c$. The cell can inspect its vicinity of radius $\mathcal{R}_N$ and can count the number $\mathcal{N}_i$ of neighboring cells. Cell age progresses with time ($\frac{d\mathcal{A}_i}{dt} = 1$), and when the cell reaches maturity, $\mathcal{A}_i \geq \mathcal{A}_i^{div}$, and is not overcrowded, that is the number of cell neighbors does not exceed the prescribed threshold, $\mathcal{N}_i \leq \mathcal{N}^{max}$, the cell will divide. Upon division of cell X_i, two daughter cells X_{i_1} and X_{i_2} are created. X_{i_1} will take position of the mother cell, i.e. $X_{i_1} = X_i$ and X_{i_2} will be located in the vicinity of the mother cell in the randomly selected direction, i.e., $X_{i_2} = X_i + ½\ \mathcal{R}_c(cos\alpha, sin\alpha)$, where $\alpha \in [0, 2\pi]$.

Each daughter cell inherits the division age from the mother cell with small noise ω, i.e., $\mathcal{A}_{i_1}^{div}, \mathcal{A}_{i_2}^{div} = \mathcal{A}_i^{div} + \omega$, where $\omega \in [-5, 5]$. The current age for each daughter cell is then set at 0, i.e., $\mathcal{A}_{i_1} = \mathcal{A}_{i_2} = 0$.

The mutation status of each daughter cell is inherited from the mother cell, i.e., $\mathcal{M}_{i_1} = \mathcal{M}_{i_2} = \mathcal{M}_i$. However, each daughter cell can acquire a new mutation status when it is born; the probability of new mutation is determined by $\mathcal{P}^{mut}$ (the only parameter we varied in the presented examples). Cell mutation will result in a shorter cell division age, i.e., $\mathcal{A}_i^{div} = ½\,\mathcal{A}^{mat}$, where $\mathcal{A}^{mat}$ is the default cell division (maturation) age. It is assumed that the random mutations will start after the cell colony reaches a noticeable size.

Cell drug absorption, accumulation, and drug-induced death

Each cell absorbs drug $\gamma(x, y, t)$ from its immediate vicinity of radius $\mathcal{R}_\gamma$. The cell uptake rate is $\mathcal{U}_\gamma$. Drug accumulation Q_i by the cell X_i is described by the following equation:

$$\frac{dQ_i}{dt} = \sum_{(x,y)} \mathcal{U}_\gamma \cdot \gamma(x, y, t)\, \chi_{\mathcal{R}_\gamma}((x, y), X_i)$$

Upon cell division, the levels of absorbed drug for both daughter cells are set to 0, i.e., $Q_{i_1} = Q_{i_2} = 0$. When the drug accumulated by a non-mutated cell ($\mathcal{M}_i = 0$) exceeds the prescribed threshold, i.e. $Q_i > Q^{thr}$, the cell will die with a probability $\mathcal{P}_Q^{die}$. Once the cell dies, it is removed from the system.

Cell random death

All cells, mutated and non-mutated, can undergo random death with a probability $\mathcal{P}^{die}$, provided that they are of considerable age, that is their current age $\mathcal{A}_i$ exceed twice the cell division age $\mathcal{A}_i^{div}$. Once the cell dies, it is removed from the system.

Cell-cell and cell-vessel interactions

Individual cells X_i and X_j can exert a cell-cell repulsive force $f_{i,j}$ (with stiffness k_c and a resting length $2\mathcal{R}_c$) to avoid overlapping:

$$f_{i,j} = \begin{cases} k_c\left(2\mathcal{R}_c - \|X_i - X_j\|\right)\dfrac{X_i - X_j}{\|X_i - X_j\|} & if\ \|X_i - X_j\| < 2\mathcal{R}_c \\ 0 & otherwise \end{cases}$$

If the cell X_i overlaps with multiple (M) cells, the cumulative repulsive force is:

$$F_i^{rep} = \sum_{n=1}^{M} k_c\left(2\mathcal{R}_c - \|X_i - X_{j_n}\|\right)\frac{X_i - X_{j_n}}{\|X_i - X_{j_n}\|}$$

To avoid overlapping between cells and vessels, a repulsive force $g_{i,j}$ (with stiffness k_v and a resting length $(\mathcal{R}_c + \mathcal{R}_v)$) between the overlapping cell X_i and the vessel, V_j is exerted on the cell:

$$g_{i,j} = \begin{cases} k_v\left((\mathcal{R}_c + \mathcal{R}_v) - \|X_i - V_j\|\right)\dfrac{X_i - V_j}{\|X_i - V_j\|} & if\ \|X_i - V_j\| < \mathcal{R}_c + \mathcal{R}_v \\ 0 & otherwise \end{cases}$$

If cell X_i overlaps with multiple (M) vessels, the cumulative repulsive force is

$$G_i^{rep} = \sum_{n=1}^{M} k_v\left((\mathcal{R}_c + \mathcal{R}_v) - \|X_i - V_{j_n}\|\right)\frac{X_i - V_{j_n}}{\|X_i - V_{j_n}\|}$$

Cell Relocation

Cell dynamics is governed by Newton's second law of motion, where the applied forces arise as a result of cell-cell repulsive interactions F_i^{rep}, repulsive interactions between cells and vessels G_i^{rep}, and forces needed to overcome medium viscosity F_i^{vis}, where m_i is cell mass:

$$m_i \frac{d^2 X_i}{dt^2} = F_i^{rep} + G_i^{rep} + F_i^{vis}$$

Assuming that springs are overdamped: $m_i \frac{d^2 X_i}{dt^2} = 0$, and that viscous force is proportional to cell velocity: $F_i^{vis} = -\nu \frac{dX_i}{dt}$, the equation of cell relocation is given by:

$$\frac{dX_i}{dt} = \frac{1}{\nu}\left(F_i^{rep} + G_i^{rep}\right)$$

All model parameters are listed in Table 1.

Table 1: Model physical and computational parameters

Parameter	Symbol	Value
**Physical parameters**		
Cell radius	$\mathcal{R}_c$	5 μm
Average cell division age	$\mathcal{A}^{mat}$	18 min
Number of neighbors for overcrowding	$\mathcal{N}^{max}$	5 cells
Cell neighborhood radius	$\mathcal{R}_N$	15 μm
Cell-cell repulsive spring stiffness	k_c	50 $\mu g/\mu m \cdot s^2$
Vessel radius	$\mathcal{R}_v$	10 μm
Cell-vessel repulsive spring stiffness	k_v	100 $\mu g/\mu m \cdot s^2$
Medium viscosity	ν	250 $\mu g/\mu m \cdot s$
Time of drug injection	$\mathcal{N}_{inj}$	250 min
Drug influx from the vessel	$\mathcal{I}_\gamma$	5 $\sigma g/\mu m^3 \cdot s$
Drug diffusion coefficient	$\mathcal{D}_\gamma$	10 $\mu m^2/s$
Drug uptake rate	$\mathcal{U}_\gamma$	0.015 s^{-1}
Cell sensing radius	$\mathcal{R}_\gamma$	3 μm
Drug level for cell killing	$\mathcal{Q}^{thr}$	4 $\sigma g/\mu m^3$
**Probability parameters**		
Probability of mutation	$\mathcal{P}^{mut}$	0.05 or 0.005
Probability of random death	$\mathcal{P}^{die}$	0.8
Probability of drug induced death	$\mathcal{P}_\mathcal{Q}^{die}$	0.85
**Computational Parameters**		
Domain size	Ω	$[-100,100]^2$ μm
Grid width	h_g	5 μm
Time step	Δt	0.25 s
Number of iterations	N^{total}	100,000
Scaling parameter	σ	10^{-19}

Output data used by the 3D lineage tree algorithms

In order to draw the 3D lineage trees, the output data needs to be prepared in the following format:

cell_history=[*cell ID*, *clone ID*, *mother ID*, *birth iter*, *div/death iter*]

cellXY_iter=[*x*, *y*]

cellID_iter=[*cell ID*]

and saved as text files: *cell_history.txt*, *cellXY_iter.txt*, and *cellID_iter.txt*, respectively, where *iter* is the iteration number. Here, *cell ID* is a unique ID number for the cell, *clone ID* is a number unique to a given clone to which the cell belongs, *mother ID* is a unique ID number of the cell's mother cell, *birth iter* is an iteration number at which the cell was born, *div/death iter* is the iteration number at which the cell either divided into two daughter cells or died, *x* and *y* are the coordinates of the cells at a given iteration *iter*. The vector *cell_history* contains history of all cells from the whole simulation and should be save at the end of simulation. The vectors *cellXY_iter* and *cellID_iter* are created and saved for the iteration with number *iter*. These data sets are initiated by: cell_history=[1,0,0,0,0], which means that the first cell has unique index 1, belongs to clone number 0, cell's mother index is 0, the cell was born in iteration 0, and is still alive (has neither died nor divided); cellXY_0=[x_0,y_0] are the coordinates of the initial cell; cellID_0=[1] is a unique index of the initial cell (first cell).

Subsequently, these data sets have to be updated in the following situations:

1. Cell division: (a) for the mother cell, find the row in *cell_history* that corresponds to the unique mother ID and update the last element to indicate the current iteration number, i.e., iteration at which the cell has divided; (b) for each daughter cell, create a new row in *cell_history* and add new unique cell ID (keep track of the last unique ID used), copy clone ID from the mother cell, copy mother cell unique ID, add the current iteration number to indicate iteration at which the cell was born, keep last element equal to 0 to indicate that the cell is alive. Repeat the same for the second daughter cell (cell ID must be unique).
2. Cell mutation: find the row in *cell_history* that corresponds to the unique cell ID and update the second component with a new unique clone ID number (keep track of the last unique clone ID used).
3. Cell random death: find the row in *cell_history* that corresponds to the unique cell ID and update the last component to indicate the iteration in which the cell died.
4. Cell drug-induced death: find the row in *cell_history* that corresponds to the unique cell ID and update the last component to indicate the iteration in which the cell died.
5. Cell removal from the system if it moved outside the computational domain: find the row in *cell_history* that corresponds to the unique cell ID and update the last component to indicate iteration in which the cell was removed.
6. Saving cell data: for those iterations *iter,* for which data is being saved in the external text files, save coordinates of all cells currently in the system (file *cellXY_iter.txt*) and unique cell IDs (file *cellID_iter.txt*) in the very same order in which the coordinates are saved.
7. Saving drug data: for the last iteration, save the values of drug concentration in all grid points (in (external text file *drug.txt*).

References

1. Berrouet C, Dorilas N, Rejniak KA, Tuncer N. Comparison of Drug Inhibitory Effects IC50 in Monolayer and Spheroid Cultures. Bull Math Biol. 2020;82(6):68. Epub 2020/06/05. doi: 10.1007/s11538-020-00746-7. PubMed PMID: 32495209.
2. Kingsley JL, Costello JR, Raghunand N, Rejniak KA. Bridging cell-scale simulations and radiologic images to explain short-time intratumoral oxygen fluctuations. PLoS Comput Biol. 2021;17(7):e1009206. Epub 2021/07/27. doi: 10.1371/journal.pcbi.1009206. PubMed PMID: 34310608; PubMed Central PMCID: PMCPMC8341701.
3. Perez-Velazquez J, Rejniak KA. Drug-Induced Resistance in Micrometastases: Analysis of Spatio-Temporal Cell Lineages. Front Physiol. 2020;11:319. Epub 2020/05/05. doi: 10.3389/fphys.2020.00319. PubMed PMID: 32362836; PubMed Central PMCID: PMCPMC7180185.
